# Sustainable bimetallic Cu/Ni catalysts: leveraging glucose for enhanced immobilization on magnetic Fe_3_O_4_/amino natural asphalt composites in coupling reactions

**DOI:** 10.1039/d5na00899a

**Published:** 2025-12-02

**Authors:** Sahar Abdolahi, Mohammad Soleiman-Beigi

**Affiliations:** a Department of Chemistry, Faculty of Basic Sciences, Ilam University P.O. Box 69315516 Ilam Iran SoleimanBeigi@yahoo.com m.soleimanbeigi@ilam.ac.ir

## Abstract

The advancement of magnetic catalytic systems utilizing natural, non-toxic precursors represents a critical need in modern science. In this study, we present an innovative protocol for synthesizing a magnetic bimetallic Cu–Ni complex on a natural asphalt support (NA-Fe_3_O_4_@glucose@Cu–Ni), in which glucose plays an important role in influencing the formation of various pentagonal and hexagonal structures in the reaction system. These geometries are likely related to the formation of glucose–metal complexation, which enhances the stability and activity of the catalyst. This catalyst was synthesized as follows: (i) nitration of natural asphalt (NA), (ii) functionalization with glucose as a bioligand, and (iii) immobilization of Cu and Ni ions to form a magnetic bimetallic complex. The catalyst was characterized by FT-IR, XRD, SEM, EDX-Map, BET, TGA, and VSM techniques. This efficient catalyst was used for the synthesis of biaryl compounds and asymmetric sulfides in polyethylene glycol (PEG) solvent at 80 °C, producing products in high yields and excellent chemical selectivity. The reactions were carried out with high efficiency and excellent chemoselectivity. Moreover, the catalyst showed remarkable reusability over six consecutive cycles with negligible loss of activity, confirming its structural integrity and long-term stability.

## Abstract 2

The advancement of magnetic catalytic systems utilizing natural, non-toxic precursors represents a critical need in modern science.

## Graphical abstract

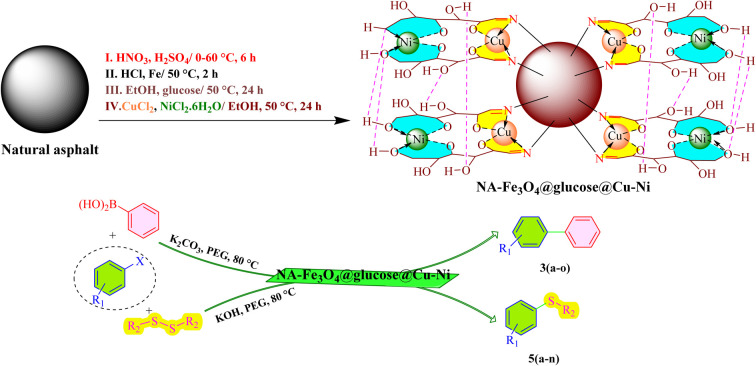

## 1. Introduction

**Fig. 1:**
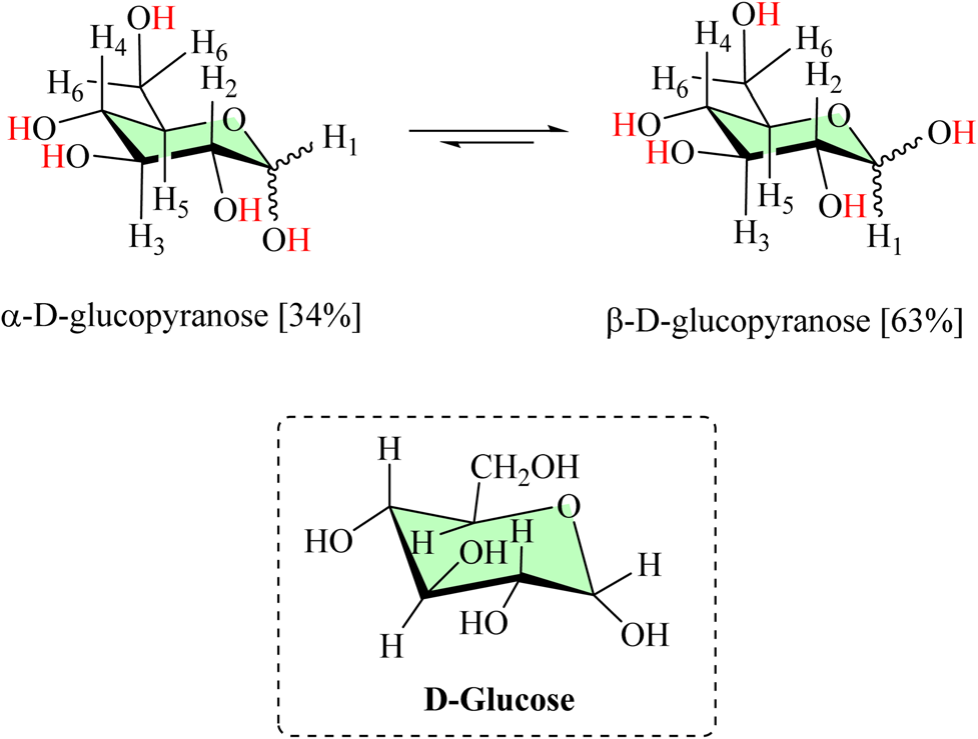
Structure of d-glucose.

Glucose, due to its versatile structure containing multiple –OH groups and oxygen atoms, can act as a natural polydentate ligand by binding to metal centers in bimetallic catalysts. This coordination not only stabilizes the active sites but also improves catalytic selectivity by providing a favorable steric arrangement. Furthermore, glucose is highly biocompatible and environmentally friendly, and is considered a sustainable alternative to conventional synthetic ligands. The equilibrium of d-glucose exists between α-d-glucopyranose and β-d-glucopyranose (Fig. 1).^1–4^ In contrast, widely utilized synthetic ligands—including triphenylphosphine (PPh_3_), bulky and electron-donating phosphines, phosphine oxides, and N-palladacycles—although highly effective in tuning the electronic and steric environment of metal centers, suffer from significant drawbacks such as high cost, toxicity, limited stability under aqueous or harsh conditions, and poor environmental compatibility.^5–7^ These limitations highlight the potential of glucose as a low-cost, green, and biocompatible ligand for catalytic applications.

Bimetallic catalytic systems, especially those involving palladium (Pd) combined with copper (Cu) or gold (Au), have been well established due to their synergistic activity and enhanced selectivity.^8–11^ However, the high cost and scarcity of Pd have directed attention toward more economical systems such as Cu/Ni. While Cu is abundant and inexpensive, its catalytic applications are limited due to aggregation, leaching, and relatively low activity. The incorporation of Ni as a secondary metal not only alleviates these drawbacks but also introduces complementary functions: Ni is efficient in activating carbon–halogen bonds, whereas Cu facilitates transmetalation and coupling steps. Therefore, the Cu/Ni bimetallic system has emerged as a promising candidate in stable catalysis.^2,11–14^ Metallic and bimetallic nanoparticles, core–shell structures, and alloys composed of metals such as Au, Ag, Cu, Co, Pd, Ni, and Ru can be synthesized *via* chemical or biological routes, using reducing agents such as monosaccharides (glucose), polysaccharides, vitamins, microwave radiation, or microorganisms. These eco-friendly approaches align with sustainable chemistry goals. Among them, Cu–Ni nanocatalysts with magnetic properties are especially attractive due to their synergistic catalytic performance and ease of recovery and reusability.^9,15–17^

Magnetic nanoparticles have attracted increasing attention as efficient and recyclable catalysts in organic synthesis due to their high surface area, ease of separation by external magnetic fields, and tunable surface chemistry. Their applications include coupling, reduction, oxidation, and multicomponent reactions, providing both catalytic efficiency and environmental stability. Recent studies have highlighted their growing role in green catalysis.^18,19^

**Fig. 2:**
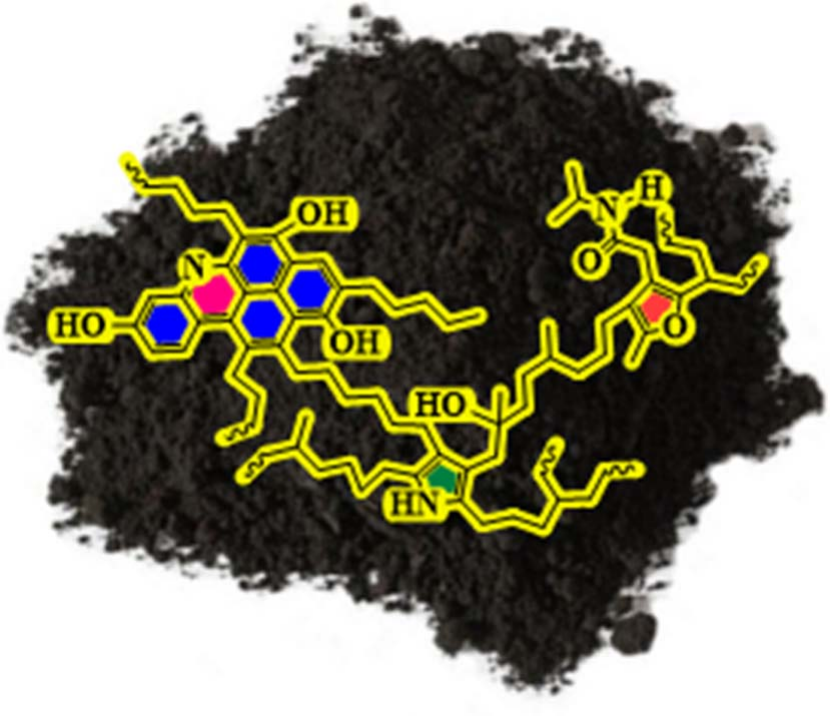
Structure of NA.

The performance of such catalysts strongly depends on their supports. Conventional carbon-based materials such as multi-walled carbon nanotubes (MWCNTs), carbon nanotubes (CNTs), graphene oxide (GO), and graphite oxide have been widely investigated. Despite their excellent physicochemical properties, their synthesis is complex, resource-intensive, and expensive. Furthermore, achieving a uniform dispersion of metal nanoparticles remains challenging, and stabilizers such as phosphines, thiols, or macrocyclic compounds—typically required for Pd—raise additional toxicity and cost issues.^20–22^ These limitations produce an urgent need for low-cost, environmentally friendly, and effective supports. Natural asphalt (NA), a natural hydrocarbon-rich material, offers significant advantages: it is abundant, durable, water-resistant, non-toxic, and cost-effective. With its high carbon content and potential for chemical modification, NA is a promising support material for nanocatalysts (Fig. 2).^23–26^

The catalytic importance of biaryl compounds and asymmetric sulfides is also significant. Biaryl scaffolds are widely found in natural products, advanced materials, and pharmaceuticals, while sulfides show significant biological and synthetic importance. Various catalytic methods—including transition metal-catalyzed cross-coupling, C–S bond formation, and metal-free oxidative coupling—have been developed for their synthesis. However, many of these methods suffer from harsh conditions, expensive catalysts, or limited recyclability, highlighting the demand for stable catalytic systems such as magnetic Cu/Ni nanocomposites.^27–29^

Therefore, in this work, an environmentally friendly Cu–Ni bimetallic nanocatalyst was developed using NA as a support and glucose as a natural bioligand. NA was chemically modified to introduce active functional groups (–NO_2_), glucose was attached to create a green stabilizing ligand, and Cu/Ni precursors (CuCl_2_/NiCl_2_·6H_2_O) were incorporated to construct a magnetic nanocatalyst with enhanced stability, activity, and recyclability. This sustainable approach provides a cost-effective and high-performance substrate for various catalytic applications.

## 2. Experimental

### 2.1. Material and apparatus

All chemicals and solvents utilized in this study were procured from Merck and Sigma-Aldrich. NA (mineral bitumen) was sourced from natural bitumen mines located in western Iran. NA was employed in its powdered form, characterized as a black solid with a melting point exceeding 240 °C and containing approximately 6% ash content. Also, carbonyl iron powder (particle size 0.5–5 µm, purity >99%) was used as the source of metallic iron powder in the synthesis. Thin layer chromatography (TLC) on Polygram SILG/UV254 silica gel plates was used to monitor the reaction progress. Fourier-transform infrared (FT-IR) spectra were recorded with a Bruker VERTEX 70 spectrometer employing the KBr pellet methodology. Morphological examination and elemental mapping (Map) were performed using a TESCAN MIRA III field emission scanning electron microscope (FE-SEM) equipped with an energy-dispersive X-ray spectrometer (EDX). X-ray diffraction (XRD) analyses were conducted utilizing a Holland Philips PW1730 diffractometer. Nitrogen adsorption–desorption isotherms at 77 K were obtained by the Brunauer–Emmett–Teller (BET) method using a Micromeritics ASAP 2020 instrument. Thermogravimetric analysis (TGA) was carried out from 25 °C to 800 °C on a NETZSCH thermogravimetric analyzer. A vibrating sample magnetometer (VSM) model MDKB was used for magnetic measurements of nanocatalysts. An atomic absorption spectroscopy (AAS) instrument, model Analytik Jena GmbH – novAA 400 P, made in Germany, was applied. Nuclear magnetic resonance (NMR) spectra, including both ^1^H-NMR and ^13^C-NMR, were recorded in CDCl_3_ using a Bruker DRX-250 AVANCE spectrometer, with tetramethylsilane (TMS) serving as an internal standard.

### 2.2. Synthesis of NA-NO_2_

NA-NO_2_ was produced using a method similar to that reported in earlier studies.^30^ For this purpose, a solution containing nitric acid/sulfuric acid (17.5/20 mL) was prepared in a 250 mL round-bottom flask. The mixture was stirred continuously at 0 °C for 15 minutes. Subsequently, 2 g of NA was gradually added to the solution. After 30 minutes, the reaction temperature was increased to 60 °C and maintained for 5 hours. Upon completion of the reaction and cooling of the mixture, 300 mL of distilled water was gradually added carefully under controlled conditions. The resulting mixture was then filtered, and the solid precipitate obtained was washed thoroughly with water. At the last stage, the product was dried in an oven at 80 °C for 3 hours, and 3 g of product was obtained.

### 2.3. Synthesis of Fe_3_O_4_@NA-NH_2_

To convert the nitro group (–NO_2_) to the corresponding amino group (–NH_2_), 15 mL of concentrated hydrochloric acid (HCl) was gradually introduced into a reaction vessel containing 2.0 g of the nitro-substituted compound and 4.0 g of Fe powder. The resulting mixture was heated to 50 °C and maintained at this temperature for 2 h to ensure complete reduction. Upon completion, the black precipitate formed was isolated by using a magnet and thoroughly washed with distilled water to remove residual acid and reaction by-products. The purified solid was obtained in a yield of 1.7 g. The reduction mechanism involves the *in situ* generation of hydrogen gas *via* the reaction of metallic iron with hydrochloric acid, which acts as the reducing agent. The black solid product exhibited complete insolubility across a broad spectrum of organic and aqueous solvents, indicating strong intermolecular forces and potential formation of an aggregated or stabilized solid matrix. Furthermore, the material demonstrated magnetic behavior, suggestive of iron oxide formation, predominantly Fe_3_O_4_, consistent with its observed physicochemical properties. This combination of solvent resistance and magnetic characteristics confers significant practical advantages, particularly for applications involving heterogeneous catalysis or environmental remediation, where facile separation, recyclability, and catalyst recovery are essential.

**Fig. 3:**
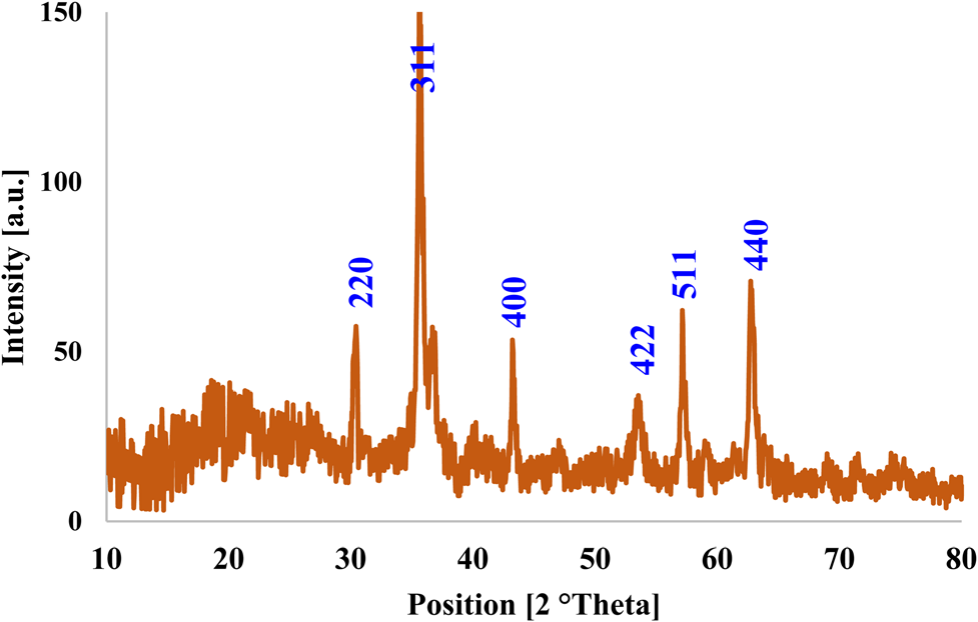
XRD pattern of Fe_3_O_4_@NA-NH_2_.

To verify the formation of Fe_3_O_4_, XRD analysis was performed. The obtained diffraction pattern showed distinct reflections consistent with the spinel structure of Fe_3_O_4_. Specifically, prominent peaks at 2*θ* values of about 30.1° (220), 35.5° (311), 43.1° (400), 53.4° (422), 57.0° (511) and 62.6° (440) were observed, which correspond to the crystallographic planes of magnetite. These diffraction peaks are well aligned with the standard reference pattern for Fe_3_O_4_ (JCPDS 01-075-0033) and confirm the successful synthesis of the target phase. Each diffraction peak corresponds to a specific set of lattice planes in the crystal structure, and correlation with the reference data provides conclusive evidence for the presence of the Fe_3_O_4_ phase (Fig. 3). In addition, the VSM of Fe_3_O_4_ was measured and the results showed that it was consistent with the studies of Fe_3_O_4_ (Fig. S1).

### 2.4. Synthesis of NA-Fe_3_O_4_@glucose

In a 100 mL flask, 0.5 g of the previously prepared Fe_3_O_4_@NA-NH_2_ and 1 g of d-glucose were combined. Subsequently, 20 mL of EtOH solvent was added to the mixture, and the reaction was allowed to proceed for 24 h at 50 °C. After the reaction was complete, the mixture was cooled and the resulting precipitate was separated using a magnet. The solid precipitate was washed multiple times with water and EtOH, resulting in a light brown powder as the final product.

### 2.5. Synthesis of NA-Fe_3_O_4_@glucose@Cu–Ni

Finally, the catalyst (NA-Fe_3_O_4_@glucose@Cu–Ni) was synthesized as follows: initially, 2.5 mmol of CuCl_2_, 1 g of NA-Fe_3_O_4_@glucose, and ethanol as the solvent in a 100 mL flask were mixed and stirred for 10 minutes. Then, 2 mmol of NiCl_2_·6H_2_O was added to the resulting mixture, and the reaction temperature was increased to 50 °C for 24 h. After the completion of the reaction, the solid product was collected using an external magnet, thoroughly washed with water several times, and dried overnight at room temperature.

**Scheme 1:**
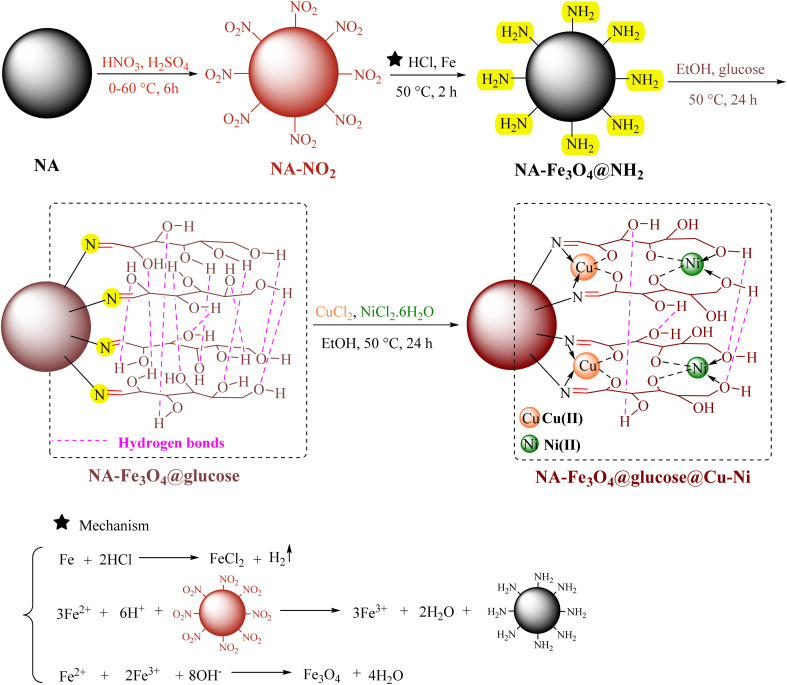
The general route for the synthesis of NA-Fe_3_O_4_@glucose@Cu–Ni.

Scheme 1 shows a bimetallic complex in which Cu and Ni are incorporated within the cyclic structures of a glucose ligand. These structures are stabilized by the NA-Fe_3_O_4_@glucose ligand, which is a multidentate ligand coordinated to both Cu and Ni. The multidentate nature of these ligands allows the formation of five- and six-membered rings, which are highly stable and robust. This feature creates stabilized and resilient cyclic structures, making the complex structure stable under various conditions and suitable for use in chemical reactions. In rings, the metals are coordinated to the –C=N groups and –OH groups, indicating the formation of a multidentate bimetallic complex.

### 2.6. General procedure for the Suzuki reaction

The Suzuki cross-coupling reaction was carried out by combining aryl halide (1 mmol), phenylboronic acid (1.2 mmol), K_2_CO_3_ (1 mmol), and 10 mg of NA-Fe_3_O_4_@glucose@Cu–Ni as a heterogeneous nanocatalyst in PEG. The mixture was stirred at 80 °C, and the reaction progress was monitored *via* TLC. Upon completion, the catalyst was recovered by using a magnet. The reaction mixture was then extracted with ethyl acetate. The crude product was purified by plate chromatography, and biphenyl derivatives were obtained in high to excellent yields.

### 2.7 General procedure for the synthesis of sulfides

A mixture of aryl halide (2.2 mmol), disulfide (1 mmol), KOH (3 mmol), and the NA-Fe_3_O_4_@glucose@Cu–Ni catalyst (20 mg) was stirred in PEG at 80 °C. The reaction progress was monitored by TLC. After completion, the catalyst was separated by using a magnet, and the product was extracted with ethyl acetate. The solvent was then evaporated, and sulfide derivatives were purified by plate chromatography, resulting in products with moderate to excellent yields.

## 3. Results and discussion

### 3.1. Characterization of NA-Fe_3_O_4_@glucose@Cu–Ni

#### 3.1.1. FT-IR analysis

**Fig. 4:**
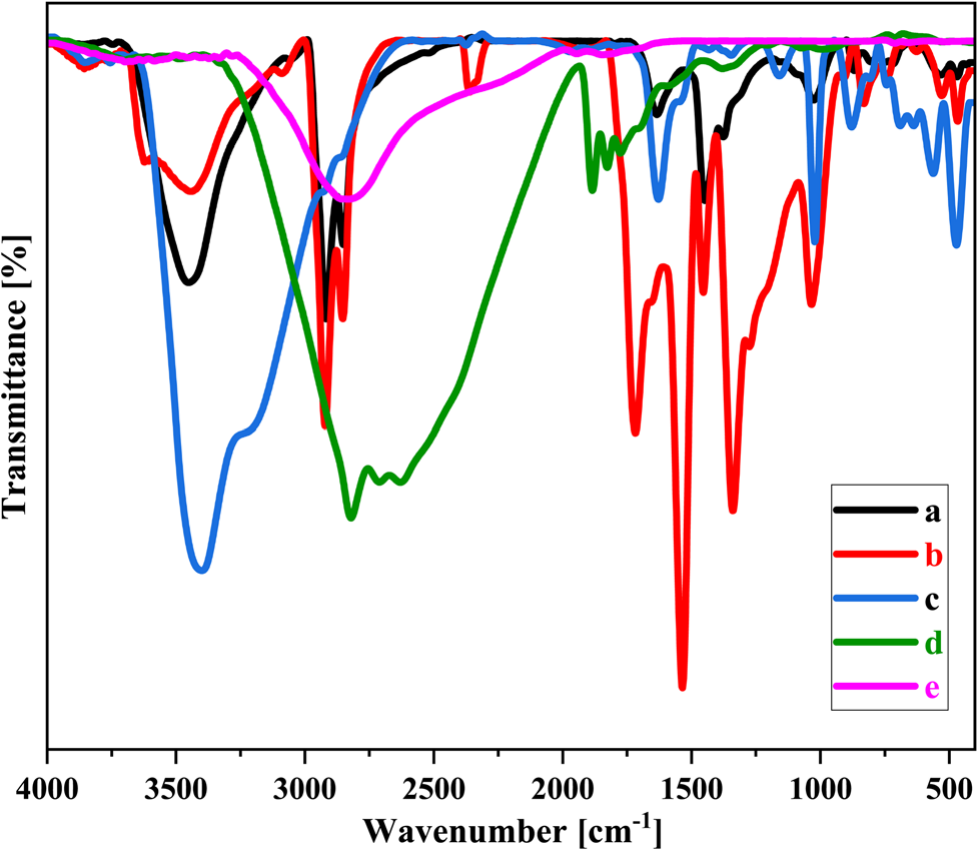
FT-IR spectra of (a) NA, (b) NA-NO_2_, (c) Fe_3_O_4_@NA-NH_2_, (d) NA-Fe_3_O_4_@glucose and (e) NA-Fe_3_O_4_@glucose@Cu–Ni.

Fig. 4a–e presents the FTIR spectra of five different compounds—NA, NA-NO_2_, Fe_3_O_4_@NA-NH_2_, NA-Fe_3_O_4_@glucose, and NA-Fe_3_O_4_@glucose@Cu–Ni—which correspond to spectra (a), (b), (c), (d), and (e), respectively. The peak observed near 3451 cm^−1^ is attributed to the stretching vibrations of both O–H and N–H bonds. The peaks observed in the range of 1449 to 1634 cm^−1^ correspond to the C=C bonds present in aromatic rings, indicating the presence of aromatic structures in NA (Fig. 4a). Additionally, the peaks appearing between 1339 and 1535 cm^−1^ are attributed to the symmetric and asymmetric stretching vibrations of –NO_2_ groups. These findings confirm that NA was effectively functionalized through the nitration process (Fig. 4b). The peaks detected at 3267 and 3405 cm^−1^ correspond to the N–H stretching vibrations of amino groups, indicating that the reduction process successfully converted nitro groups into amino groups, thereby introducing –NH_2_ functionalities into the structure. Furthermore, the peak at 1158 cm^−1^ is assigned to the C–N stretching vibration, which is characteristic of amine groups. The presence of this peak further confirms the formation of amino groups during the reduction process (Fig. 4c). The reduction reaction using HCl and Fe has caused notable changes in the material's chemical composition, as shown in Fig. 4c. One important observation is the appearance of a peak at 582 cm^−1^, which is assigned to the Fe–O bond. In the 4d spectrum, a new peak appears at 1629 cm^−1^. This change indicates the formation of a –C=N bond, resulting from the chemical reaction between the –NH_2_ groups on the surface of Fe_3_O_4_@NA-NH_2_ and the C=O groups present in glucose. This new peak is also confirmed when compared to the spectrum of pure glucose (Fig. S2). In this spectrum, absorption bands appear in the wavenumber range of around 2500 to 3700 cm^−1^, which include two broad overlapping bands attributed to the stretching vibrations of O–H and C–H bonds. These bands are influenced by hydrogen bonding, which affects the O–H stretching frequencies. The O–H vibrations can originate from both primary and secondary alcohol groups. Absorptions in the region of 3200 to 3500 cm^−1^ are mainly assigned to O–H stretching vibrations and likely represent twin peaks from both primary and secondary alcohol groups. Meanwhile, absorptions between 2800 and 3000 cm^−1^ are primarily due to C–H stretching vibrations. The C–O stretching vibrations from primary and secondary alcohol groups are found in the range of nearly 1000 to 1150 cm^−1^.

The stepwise FT-IR spectra have been provided separately in the SI (Fig. S3–S7).

The FTIR spectrum of NA-Fe_3_O_4_@glucose@Cu–Ni (Fig. 4e) provides clear evidence of the successful coordination between NA-Fe_3_O_4_@glucose and the metal centers. The shifts observed at 1621 and 3416 cm^−1^ indicate that the functional groups associated with these vibrations, such as –C=N and OH groups, are affected by the complexation process. These frequency changes arise from the interaction of the multidentate ligand (NA-Fe_3_O_4_@glucose) with Cu and Ni ions, leading to the formation of a stable Cu–Ni bimetallic complex. In other words, the ligands on the surface of NA-Fe_3_O_4_, including amine and glucose groups, act as electron donors and bind to the metal ions, resulting in a well-organized and stable complex structure. These results confirm the successful synthesis of the Cu–Ni bimetallic complex and highlight the important role of the identified functional groups in stabilizing the complex.

#### 3.1.2. XRD analysis

**Fig. 5:**
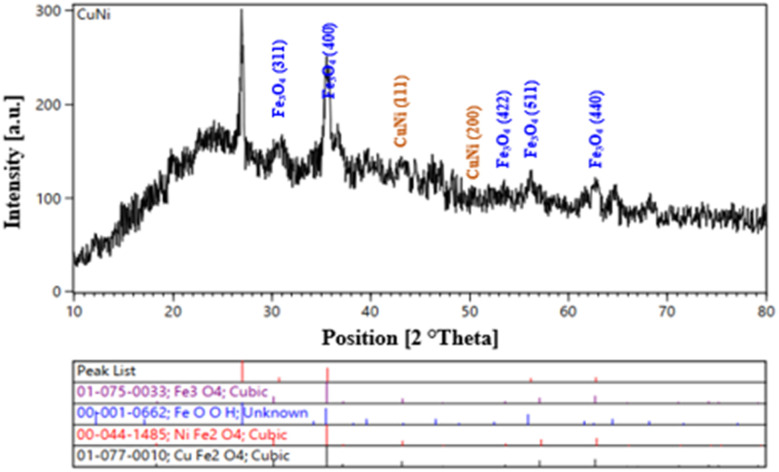
XRD pattern of NA-Fe_3_O_4_@glucose@Cu–Ni.

In Fig. 5, the 2*θ* peaks at Fe_3_O_4_ (311) ≈ 30.1°, (400) ≈ 35.5°, (422) ≈ 53.1°, (511) ≈ 57.0°, and (440) ≈ 62.5° indicate the presence of the magnetic spinel Fe_3_O_4_ phase, with NiFe_2_O_4_ and CuFe_2_O_4_ also observed, confirming the coexistence of spinel ferrite phases within NA-Fe_3_O_4_@glucose@Cu–Ni. The FCC reflections corresponding to Cu and Ni or Cu/Ni alloys appear in the ∼43–74° range, depending on the alloy composition. The simultaneous presence of spinel magnetic phases and FCC Cu/Ni phases demonstrates the nanostructured bi-phasic coexistence and synergistic enhancements in catalytic and magnetic properties, supported by Table S1 and standard references (ICDD/JCPDS).

#### 3.1.3. SEM analysis

**Fig. 6:**
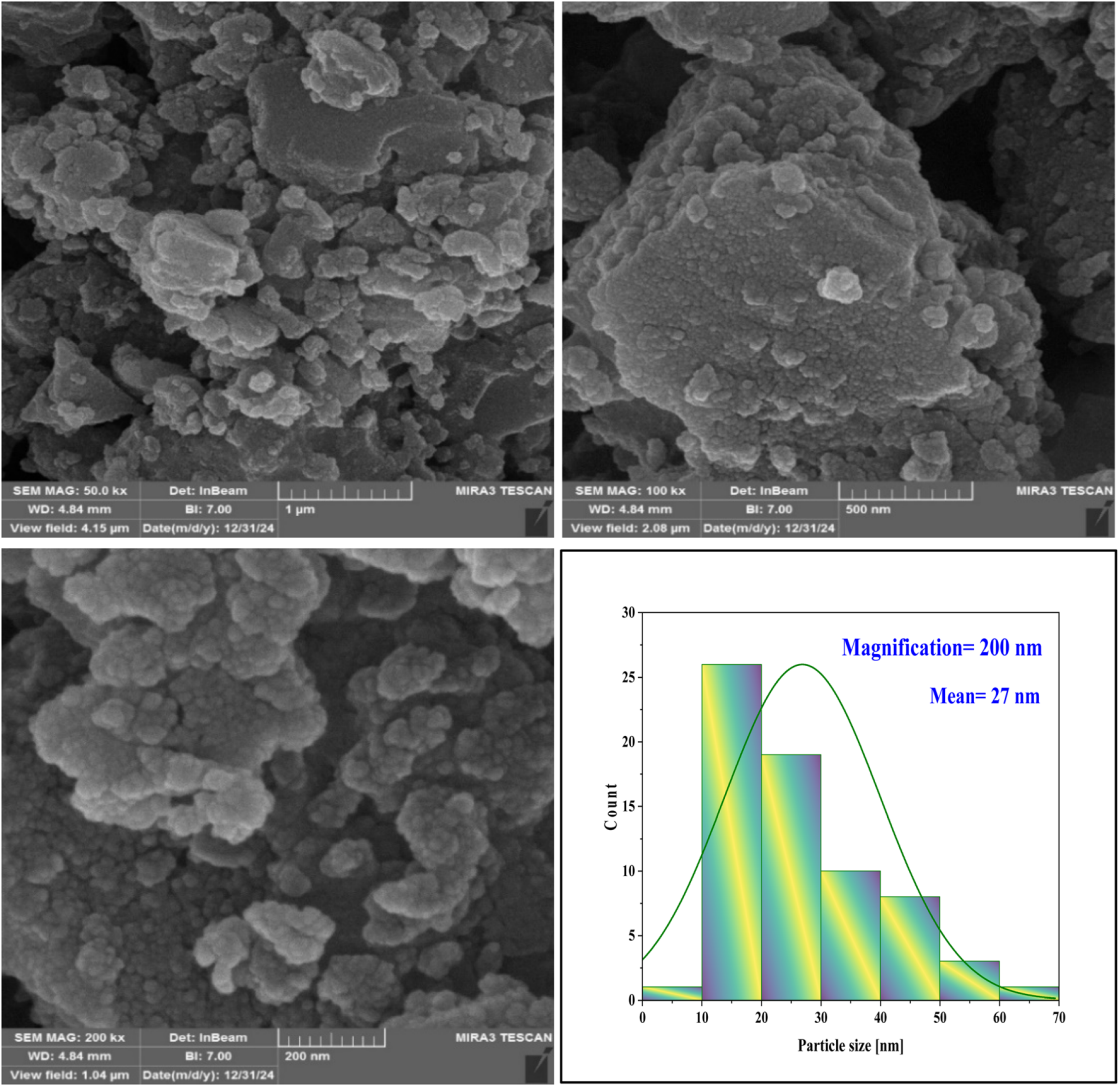
SEM images and histograms of NA-Fe_3_O_4_@glucose@Cu–Ni.

SEM images of NA-Fe_3_O_4_@glucose@Cu–Ni and its histogram are shown in Fig. 6. The SEM images reveal the surface morphology of the Cu–Ni bimetallic magnetic nanocatalyst, exhibiting an aggregated and clustered appearance. The nanoparticles are predominantly observed as dense, spherical clusters, indicating that the synthesis process has led to the agglomeration of nanosized particles. Small particles are gathered inside the clusters, forming bulk and polyhedral structures. This suggests that the distribution of Cu and Ni elements on the sample surface predominantly occurs in the form of dense clusters and agglomerates. The surface of these particles appears rough and irregular, featuring various protrusions and pores. Such surface characteristics contribute to an increased active surface area and enhance surface contact during catalytic processes. The particle density and the presence of surface porosity further improve the accessible surface area, facilitating easier interaction between reactive species and the nanoparticles. Consequently, this aggregated and clustered morphology enhances the magnetic and catalytic properties of the nanocatalyst, ultimately improving its efficiency and performance in relevant applications.

The histogram analysis reveals that over half of the nanoparticles fall within the 10 to 30 nanometer size range, with an average size of approximately 27 nm. This suggests that the synthesis process predominantly yields smaller-sized nanoparticles. In general, the size distribution indicates that most particles fall within the nanometer range, although some variation and diversity in particle sizes are present. This dispersion suggests that the synthesis process produces particles with varying diameters, yet the average size remains around 27 nm. Such a distribution is highly suitable for nanocatalyst applications, as it demonstrates reasonable control over particle size and flexibility in tuning the final structure.

#### 3.1.4. EDX-Map analysis

**Fig. 7:**
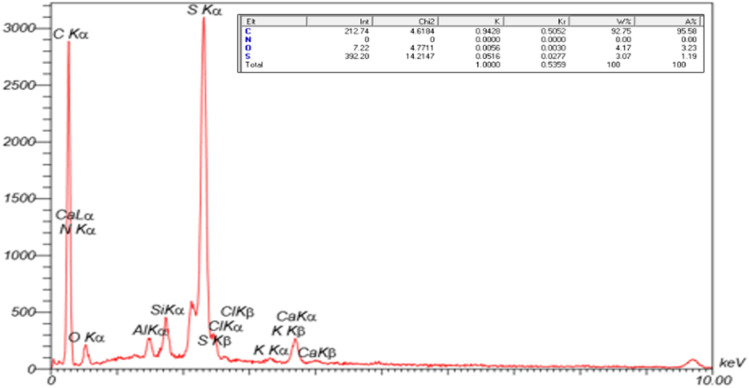
EDX analysis of NA.

The EDX spectrum of NA shows that carbon (C ≈ 93 wt%) and sulfur (S ≈ 4 wt%) are the dominant elements, accompanied by minor peaks corresponding to oxygen, silicon, aluminum, chlorine, and calcium. These elements are attributed to the mineral and inorganic impurities naturally present in raw asphalt. However, due to the inherently complex and heterogeneous composition of natural asphalt, it is difficult to accurately identify and confirm the presence of all elemental species in its structure (Fig. 7).

**Fig. 8:**
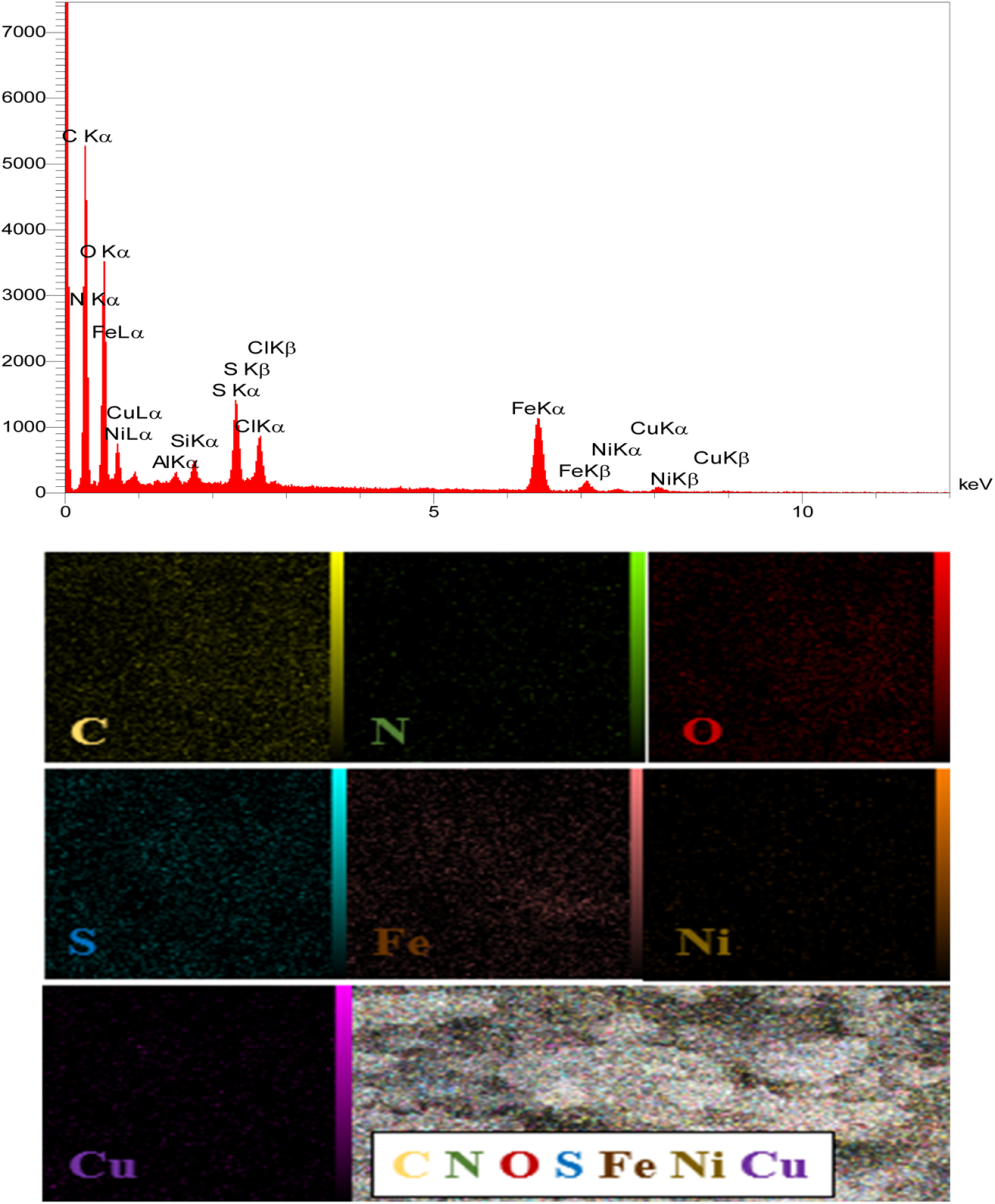
EDX mapping analysis of NA-Fe_3_O_4_@glucose@Cu–Ni.

In contrast, the EDX spectrum of the Cu–Ni magnetic nanocatalyst shows strong and well-defined peaks of Fe, Cu, and Ni, confirming the successful incorporation of these metal components. The quantitative EDX data clearly show that the relative percentage of auxiliary elements such as Al, Si and Cl in the final nanocatalyst is significantly reduced compared to the raw asphalt, indicating their minor residual presence as minor impurities from the natural matrix or precursor salts. Therefore, the results demonstrate a relatively uniform elemental distribution, which can contribute to enhanced magnetic and catalytic properties (Fig. 8).

#### 3.1.5. BET analysis

**Fig. 9:**
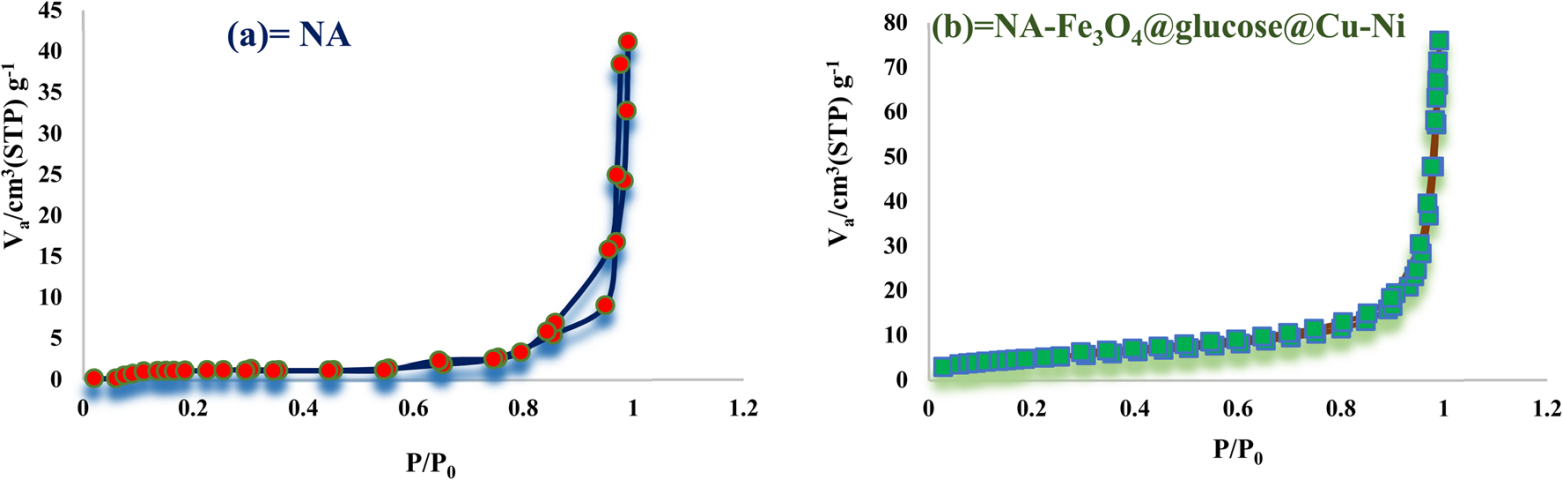
N_2_ adsorption–desorption isotherms of (a) NA and (b) NA-Fe_3_O_4_@glucose@Cu–Ni.

The specific surface area, volume, and pore diameter of NA-Fe_3_O_4_@glucose@Cu–Ni were determined using BET analysis. According to the IUPAC classification, the magnetic Cu–Ni bimetallic complex exhibited a type III isotherm. This isotherm type is typically associated with microporous materials, which fall within the category of nanomaterials. Based on the BET results, the surface area, pore volume, and pore diameter of the nanocatalyst were found to be 16.75 m^2^ g^−1^, 0.111 cm^3^ g^−1^, and 2 nm, respectively. In comparison, these values for NA were measured to be 10.49 m^2^ g^−1^, 0.149 cm^3^ g^−1^, and 10 nm, respectively. These findings indicate that the reduction in particle size leads to an increase in surface area, highlighting the significant differences between the Cu–Ni bimetallic complex and the NA support (Fig. 9).

#### 3.1.6. TGA

**Fig. 10:**
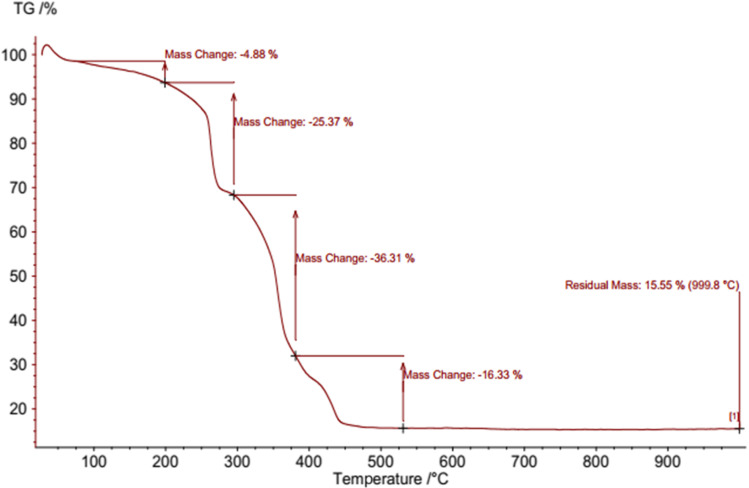
TG curve of NA-Fe_3_O_4_@glucose@Cu–Ni.

The TG curve reveals multiple stages of weight loss, reflecting various processes that occur as the sample is heated. The initial weight loss typically takes place at lower temperatures, approximately at 25–200 °C, and is attributed to the evaporation of water and organic solvents contained within the sample. This stage is usually rapid and well-defined because these volatile substances readily vaporize, causing a decrease in the sample's mass. At higher temperatures, generally ranging from 200 to 400 °C and above, further weight loss occurs due to the decomposition of organic components, surface oxidation, and other chemical changes within the nanocatalyst. These latter processes tend to occur more gradually, resulting in a slower and more continuous mass reduction. During this phase, organic materials and surface-bound species break down and oxidize, contributing to the ongoing decrease in the sample's weight (Fig. 10).

#### 3.1.7. VSM analysis

**Fig. 11:**
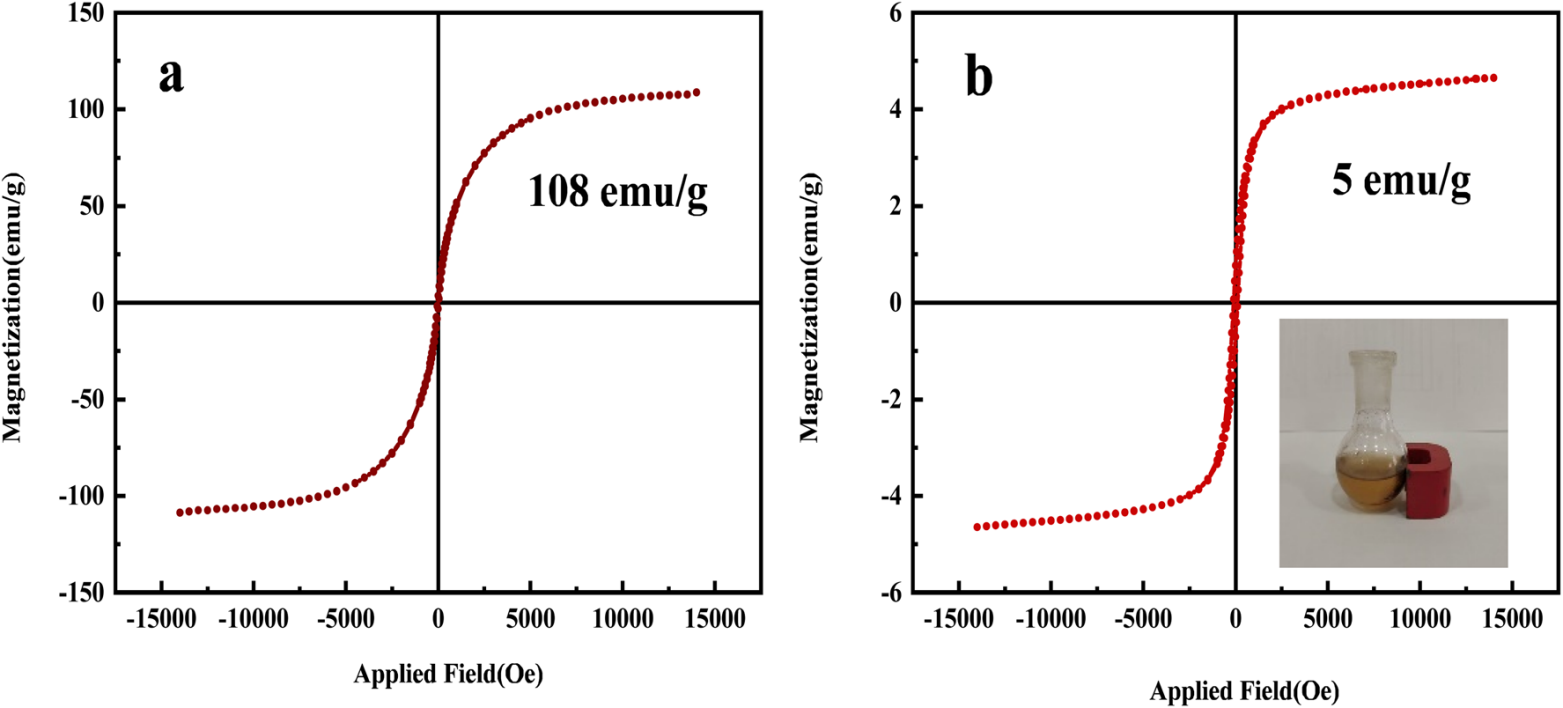
VSM diagram of (a) NA-Fe_3_O_4_@glucose and (b) NA-Fe_3_O_4_@glucose@Cu–Ni. The slight increase in *M*_s_—from 104 emu g^−1^ for Fe_3_O_4_@NA-NH_2_ to 108 emu g^−1^ for NA-Fe_3_O_4_@glucose—can be explained by improved dispersion of the Fe_3_O_4_ nanoparticles after glucose functionalization, which reduces aggregation and strengthens interparticle magnetic coupling. Moreover, the glucose layer may partially passivate surface defects and better preserve the magnetic core, yielding a slight net increase in *M*_s_. The small magnitude of this change indicates that the Fe_3_O_4_ core structure is retained and that the organic coating does not compromise the material's magnetic behavior.

As shown in Fig. 11, the magnetic properties of two samples, NA-Fe_3_O_4_@glucose (Fig. 10a) and the bimetallic complex NA-Fe_3_O_4_@glucose@Cu–Ni (Fig. 10b), were investigated using a vibrating sample magnetometer (VSM). The saturation magnetization (*M*_s_) of the NA-Fe_3_O_4_@glucose ligand was approximately 108 emu g^−1^, indicating strong magnetic properties due to the presence of Fe_3_O_4_ nanoparticles. However, upon addition of Cu and Ni metal ions, the saturation magnetization decreased to about 5 emu g^−1^. This reduction is attributed to the presence of diamagnetic organic ligands and the combination of ferromagnetic and diamagnetic metals in the complex structure, which weakens the overall magnetic moment of the system. Nevertheless, the bimetallic sample still retains sufficient magnetization for easy magnetic separation and catalyst recovery, a feature that is highly important for practical applications.

#### 3.1.8. AAS analysis

Based on AAS analysis, the Cu and Ni contents in NA-Fe_3_O_4_@oxalic acid@Cu(ii) were 0.27 and 0.14 mmol g^−1^, respectively. Such amounts of Cu and Ni are expected to influence the catalytic performance significantly: Cu can provide active sites for electron transfer, while Ni may contribute to modifying the pore structure or enhancing the stability of the magnetic framework. Together, these metals improve the accessibility of reactants to the active sites, facilitating more efficient catalysis and higher turnover numbers.

### 3.2. Catalytic activity

Following the successful synthesis and characterization of NA-Fe_3_O_4_@glucose@Cu–Ni, the catalytic performance of this nanocomposite was evaluated in C–C and C–S coupling reactions. The coupling between iodobenzene and phenylboronic acid to form product 3a was chosen as a model reaction for optimizing the reaction conditions. Key parameters, including catalyst amounts, solvent type, temperature, and base amount, were investigated. Under fixed conditions—constant solvent, temperature, base, and substrate concentrations—the model reaction was carried out with varying amounts of the NA-Fe_3_O_4_@glucose@Cu–Ni nanocatalyst. Reaction progress was monitored at regular intervals using TLC. In the absence of the catalyst, no product formation was observed, confirming the catalytic necessity of the nanocomposite. Optimal catalytic activity was achieved using 10 mg of NA-Fe_3_O_4_@glucose@Cu–Ni in PEG. Subsequent screening of solvents, bases, and temperatures revealed that PEG as the solvent and K_2_CO_3_ as the base provided superior results. Optimization of different amounts of bases also showed that the same 1 mmol of K_2_CO_3_ gave the best yield (Table S2). The highest yield was obtained in PEG with 10 mg of the catalyst and 1 mmol of K_2_CO_3_ at 80 °C for 8 minutes (Table 1, entry 3).

**Table 1:** Optimization of reaction parameters for the synthesis of 1,1′-biphenyl using NA-Fe_3_O_4_@glucose@Cu–Ni^a^

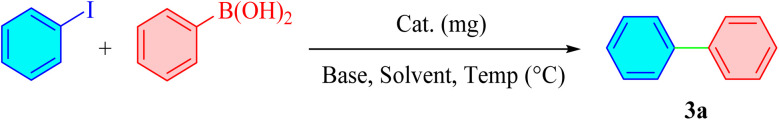					
Entry	Base	Temperature (°C)	Solvent	Amount of catalyst (mg)	Yield^b^ (%)
1	K_2_CO_3_	80	PEG	—	NP^c^
2	K_2_CO_3_	80	PEG	5	90
**3**	**K**_**2**_ **CO**_**3**_	**80**	**PEG**	**10**	**99**
4	K_2_CO_3_	80	PEG	15	97
5	K_2_CO_3_	50	PEG	10	92
6	Ca_2_CO_3_	80	PEG	10	93
7	Na_2_CO_3_	80	PEG	10	94
8	Cs_2_CO_3_	80	PEG	10	90
9	NaOH	80	PEG	10	91
10	KOH	80	PEG	10	92
11	K_2_CO_3_	Reflux	H_2_O	10	88
12	K_2_CO_3_	Reflux	EtOH	10	85
13	K_2_CO_3_	80	DMF	10	90

a Reaction conditions: iodobenzene (1 mmol), phenylboronic acid (1.2 mmol), base (1 mmol), NA-Fe_3_O_4_@glucose@Cu–Ni catalyst (mg), and solvent (1 mL) for 8 minutes.

b Isolated yields.

c No product.

Based on the optimized conditions presented in Table 1, the scope of the reaction was examined using a variety of aryl halides, including those bearing electron-donating and electron-withdrawing substituents, alongside phenylboronic acid. This led to the successful synthesis of 3(a–o) biphenyl derivatives. The data indicated that aryl iodides exhibited higher reactivity compared to aryl bromides and aryl chlorides. To evaluate the chemoselectivity of the novel catalytic system, reactions involving 1-chloro-4-iodobenzene and 1-bromo-4-iodobenzene with phenylboronic acid were conducted. In both cases, the bromide and iodide substituents exhibited higher reactivity compared to the chloro groups (Table 2, 3h and 3i).^31,32^

**Table 2:** Suzuki synthesis of 1,1′-biphenyl derivatives catalyzed by NA-Fe_3_O_4_@glucose@Cu–Ni^a^^,^^b^

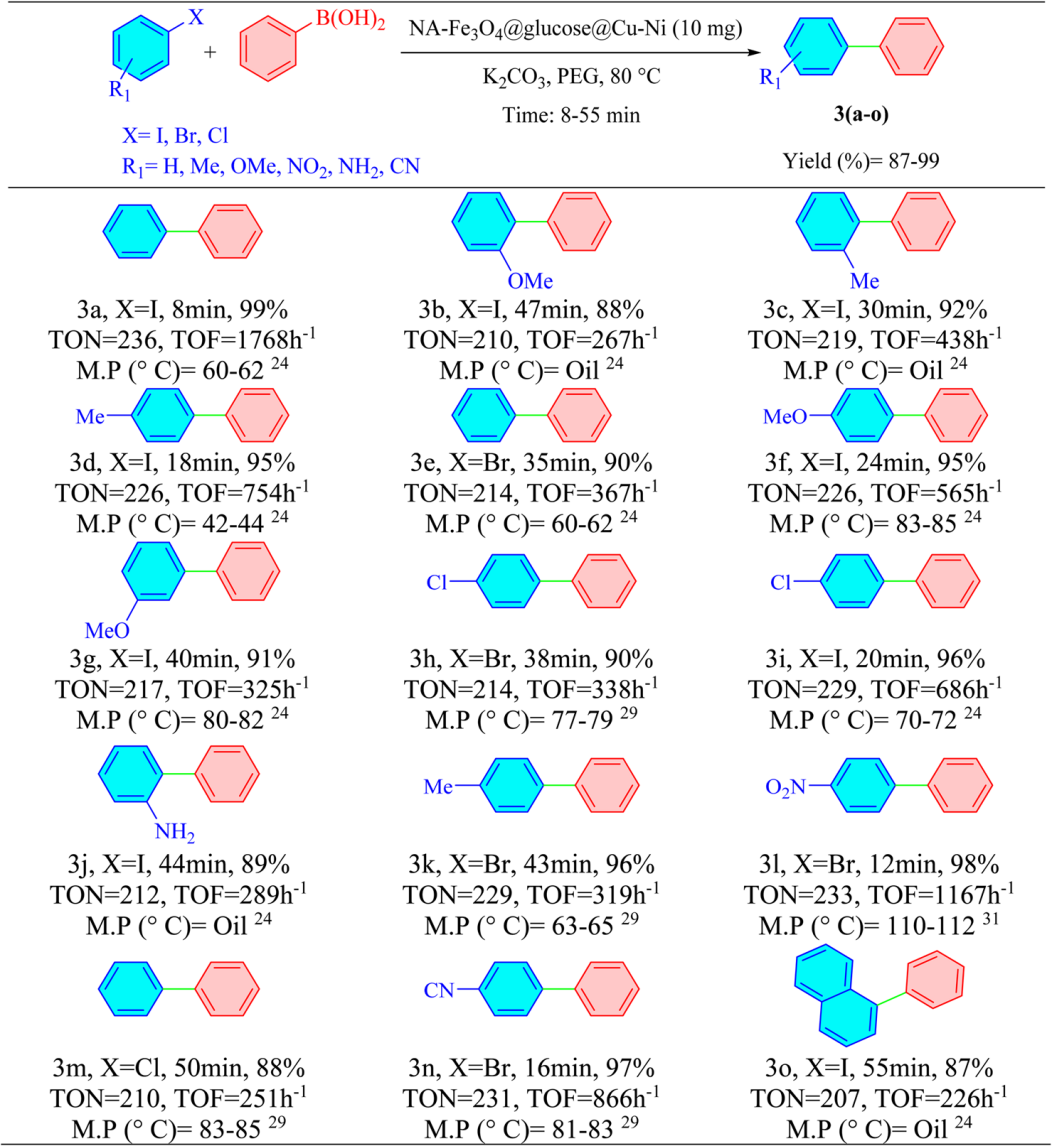

a Reaction conditions: aryl halide (1 mmol), phenylboronic acid (1.2 mmol), K_2_CO_3_ (1 mmol), and NA-Fe_3_O_4_@glucose@Cu–Ni (10 mg) at 80 °C in PEG (1 mL).

b Isolated yield.

The efficiency of NA-Fe_3_O_4_@glucose@Cu–Ni as a catalyst was evaluated in C–S bond formation reactions. For this investigation, iodobenzene and diphenyl disulfide were employed as model reactants to form product 5a. The results from experiments clearly indicated that no product was formed in the absence of the catalyst (Table 3, entry 1), underscoring its essential role in driving the reaction forward. Optimization experiments revealed that the optimal amount of catalyst was 20 mg, which afforded a maximum product yield of 98%. When evaluating different solvents, PEG emerged as the most effective medium, likely due to its excellent phase-transfer capabilities. Among the bases tested, KOH delivered the best results. The optimization of base quantities showed that the best yield was obtained using 3 mmol of KOH (Table S3). Temperature also had a significant effect on the reaction outcome. Although product formation was observed at various temperatures, complete conversion was only achieved at 80 °C, making it the most favorable condition. In summary, the optimal conditions for synthesizing asymmetric sulfides involved using 20 mg of NA-Fe_3_O_4_@glucose@Cu–Ni, KOH as the base, and PEG as the solvent at a temperature of 80 °C (Table 3, entry 5).

**Table 3:** Optimization of reaction parameters for the synthesis of sulfides using NA-Fe_3_O_4_@glucose@Cu–Ni^a^

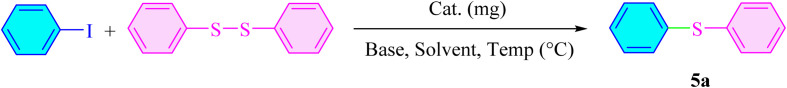					
Entry	Base	Temperature (°C)	Solvent	Amount of catalyst (mg)	Yield^b^ (%)
1	KOH	80	PEG	—	NP^c^
2	KOH	80	PEG	5	90
3	KOH	80	PEG	10	93
4	KOH	80	PEG	15	96
**5**	**KOH**	**80**	**PEG**	**20**	**98**
6	KOH	80	PEG	25	98
7	Cs_2_CO_3_	50	PEG	20	90
8	NaOH	80	PEG	20	97
9	Na_2_CO_3_	80	PEG	20	91
10	K_2_CO_3_	80	PEG	20	92
11	KOH	Reflux	H_2_O	20	90
12	KOH	Reflux	EtOH	20	88
13	KOH	80	DMSO	20	97
14	KOH	80	Toluene	20	70
15	KOH	80	DMF	20	94

a Reaction conditions: iodobenzene (2.2 mmol), diphenyl disulfide (1 mmol), base (3 mmol), NA-Fe_3_O_4_@glucose@Cu–Ni catalyst (mg), and solvent (1 mL) for 18 minutes.

b Isolated yields.

c No product.

After establishing the optimized conditions, various aryl halides bearing electron-donating and electron-withdrawing substituents were evaluated in the presence of disulfides. This investigation successfully synthesized a range of 5(a–n) asymmetric sulfides. The findings revealed that aryl iodides demonstrated greater reactivity compared to aryl bromides and chlorides. For this purpose, to assess the chemoselectivity of the catalyst in the synthesis of asymmetric sulfides, reactions involving 1-iodo-4-chlorobenzene and 1-iodo-4-bromobenzene were conducted. In both cases, the iodide substituent exhibited higher reactivity compared to the chloro and bromo groups (Table 4, 5f and 5l).

**Table 4:** Synthesis of asymmetric sulfide derivatives catalyzed by NA-Fe_3_O_4_@glucose@Cu–Ni^a^^,^^b^

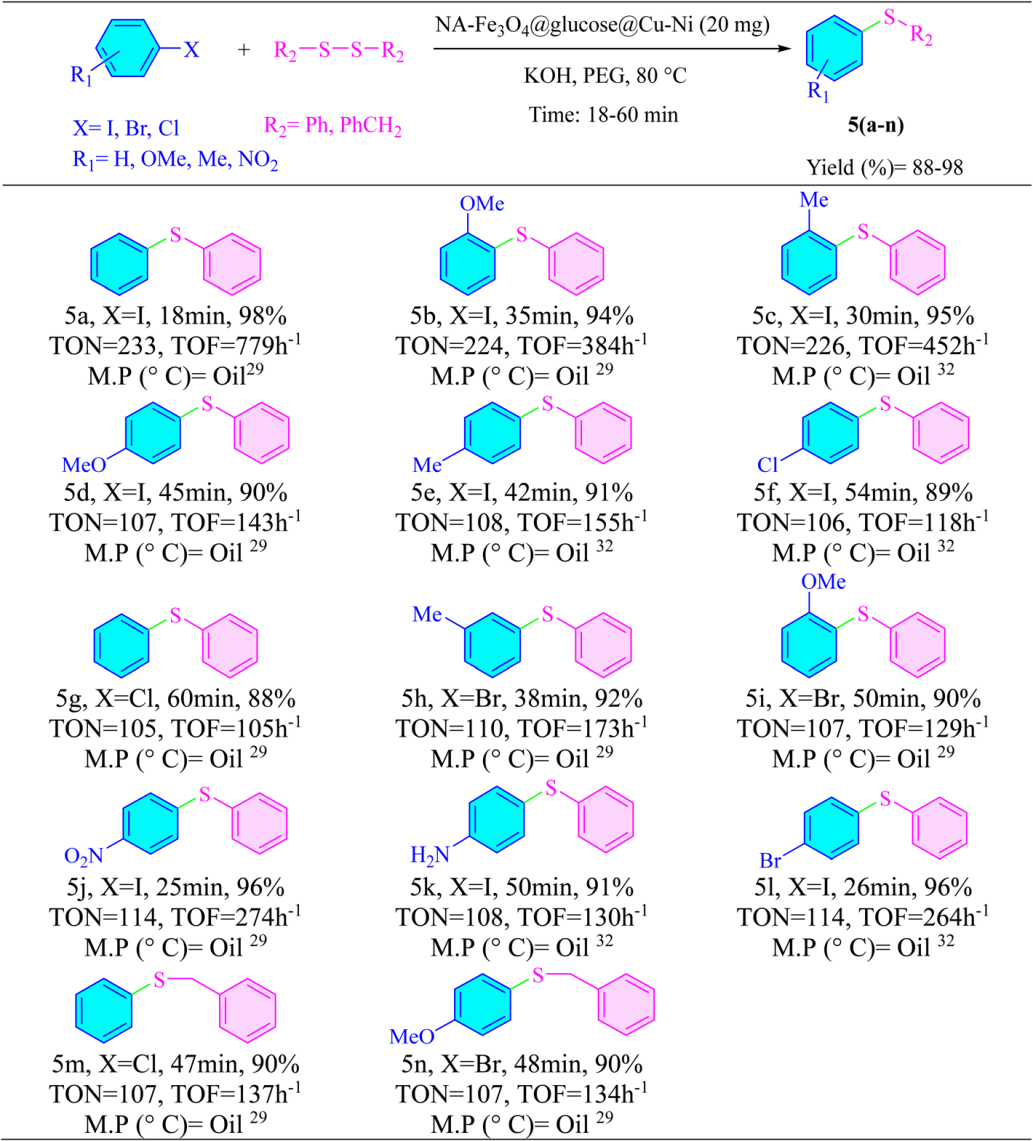

a Reaction conditions: aryl halide (2.2 mmol), disulfide (1 mmol), KOH (3 mmol), and NA-Fe_3_O_4_@glucose@Cu–Ni (20 mg) at 80 °C in PEG (1 mL).

b Isolated yield.

Further verification of the product formation was achieved by performing ^1^H-NMR and ^13^C-NMR analyses on multiple samples, with the related data exhibited in Fig. S8–S33.

### 3.3. Catalyst recycling and reusability

**Fig. 12:**
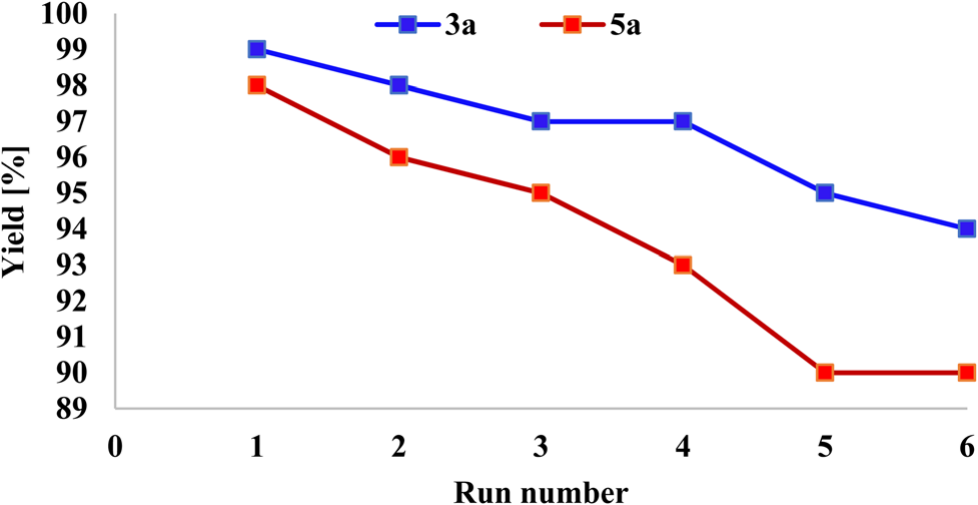
Reusability of NA-Fe_3_O_4_@glucose@Cu–Ni for the synthesis of products 3a and 5a.

As shown in Fig. 12, a comparative analysis between the fresh and reused catalyst confirmed the structural stability of the catalyst after multiple cycles. Notably, the catalyst was able to retain its catalytic efficiency over six consecutive cycles for 3a and 5a without significant loss of activity. This high recyclability reflects the robust nature of the catalyst's structure and surface properties. Moreover, the magnetic nature of the catalyst allows for its simple and rapid separation from the reaction mixture using an external magnet, eliminating the need for energy-intensive filtration or centrifugation steps. This aligns with the principles of green chemistry by promoting resource efficiency, reducing chemical waste, and minimizing environmental impact, making the process both sustainable and economically advantageous.

**Fig. 13:**
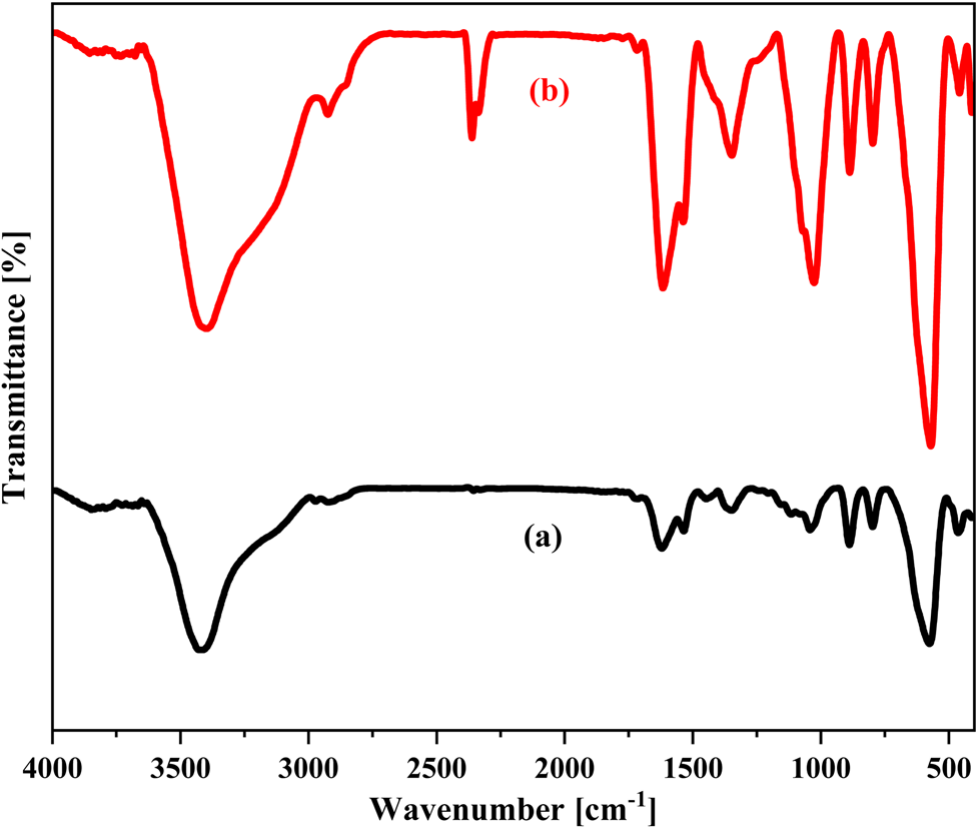
The FT-IR spectra of (a) fresh NA-Fe_3_O_4_@glucose@Cu–Ni and (b) NA-Fe_3_O_4_@glucose@Cu–Ni after 6 runs for compound 3a.

Furthermore, the FT-IR spectrum of the catalyst after six recovery cycles for compound 3a showed no significant shifts or displacement in the characteristic peaks. This confirms that the catalyst has retained its structural stability throughout repeated usage (Fig. 13).

**Fig. 14:**
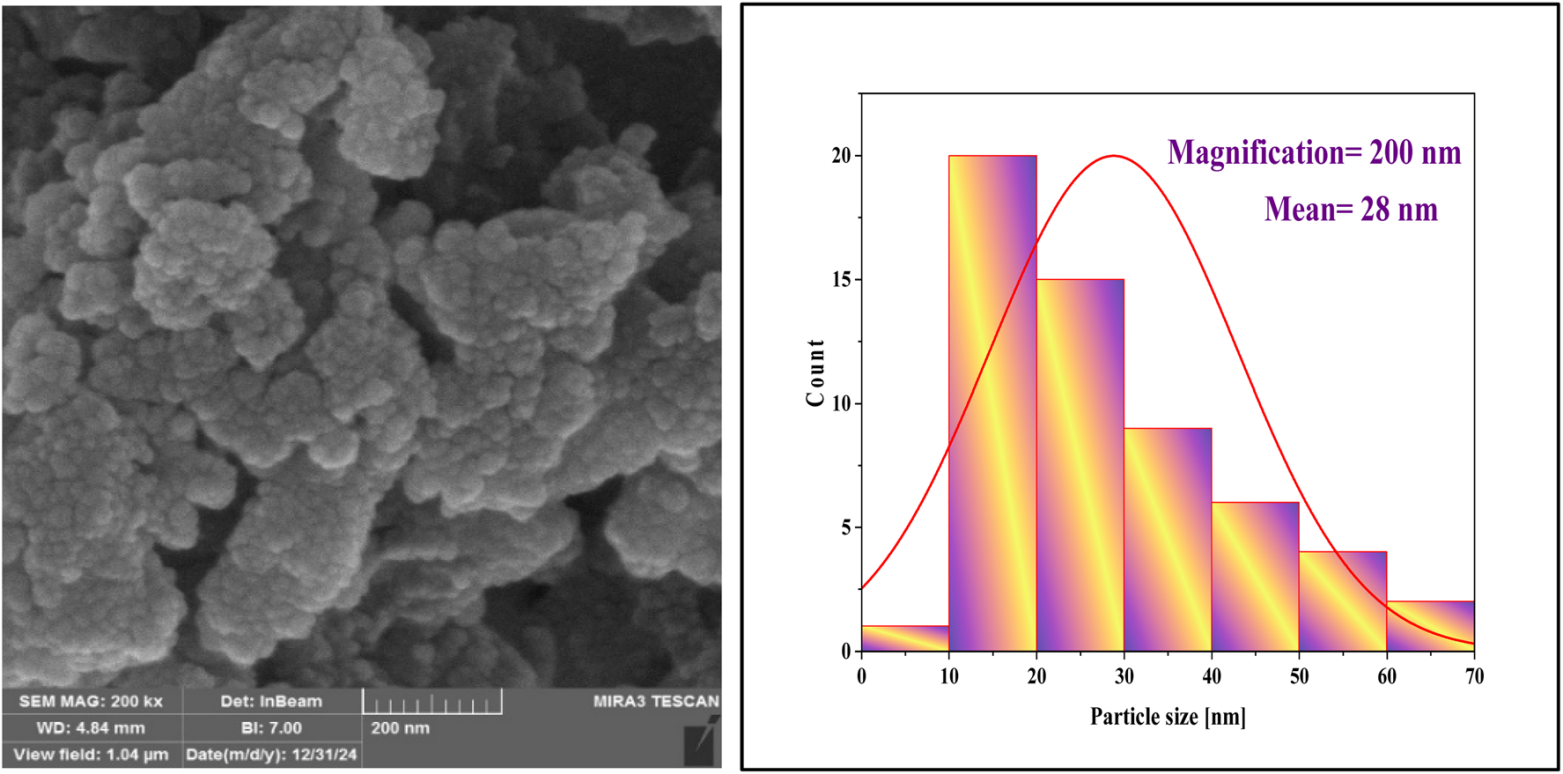
SEM image and histogram of NA-Fe_3_O_4_@glucose@Cu–Ni after 6 cycles for compound 3a.

In addition, the SEM images and histogram of the recycled catalyst show that after six cycles of reaction or use for compound 3a, the catalyst's morphology and appearance have not undergone significant changes. This is important because maintaining the structure and physical properties of the catalyst over time indicates a high resistance to wear or degradation, which implies a stable performance and a long useful lifespan. Therefore, the catalyst is capable of functioning effectively in repeated processes without a loss of activity (Fig. 14).

### 3.4. Hot filtration

To evaluate the heterogeneity of the catalyst and assess potential leaching of active sites into the reaction medium, a hot filtration test was conducted using the model reaction between iodobenzene and phenylboronic acid for compound 3a. After half of the total reaction time had elapsed, the product was isolated with a 53% yield. The experiment was then repeated, but this time the magnetic catalyst was removed from the reaction mixture halfway through the reaction by applying an external magnet. The filtrate solution was subsequently allowed to react under the same reaction conditions. The reaction continued, yielding 57% of the desired product. These findings demonstrate that the catalyst's active Cu and Ni sites did not leach into the solution during the reaction. Furthermore, the results confirm that the Cu–Ni bimetallic catalyst retained its structural stability throughout the process.

### 3.5. Comparison

In many recent catalytic studies, advanced and expensive carbon-based materials such as MWCNTs, CNTs, GO, and graphite oxide have been widely used. These materials often require complex and costly synthesis procedures, and achieving uniform dispersion of metal nanoparticles on their surfaces is challenging. Additionally, the stabilization of active metals like Pd is typically achieved using toxic and non-biodegradable ligands such as phosphines, thiols, or complex macrocyclic compounds. These ligands not only pose environmental and health risks but also suffer from high production costs and limited stability under specific reaction conditions.^20–22^ In contrast, the use of NA as a carbonaceous support offers a highly attractive alternative due to its low cost, abundance, ease of availability, and chemically rich and diverse structure, making it well-suited for the stabilization of nanocatalysts. Furthermore, the use of glucose as a biocompatible, non-toxic, and biodegradable ligand provides a green and safe replacement for conventional organic ligands. With its multiple hydroxyl groups, glucose shows strong coordination ability with active metals such as Cu and Ni. This enables effective metal stabilization on the support surface, prevents nanoparticle agglomeration, and minimizes unwanted metal leaching during catalytic reactions (Table 5).

**Table 5:** Comparison of NA with other carbon nanomaterials and ligand effects on metal catalysis: materials, applications, and limitations

Raw	Carbon material	Ligand type	Metal complex/catalyst	Catalytic application	Disadvantages of the carbon material/ligand	Ref.
1	MWCNT	Macrocyclic pyrimidine	Pd(ii) complex	ORR^a^	High cost; limited dispersion; possible toxic ligand residues on product	33
2	MWCNT/graphene/GNPT^c^	Macrocyclic^b^	Pd(ii) non-covalent complex on surface	Cu-free Sonogashira (≥90%)	Lower adsorption on CNTs; reduced cycle efficiency on MWCNTs; costly	34
3	Graphene (flat surface)	Non-covalent (π–π)	Pd(ii) complex	Suzuki	Requires precise experimental conditions; uneven dispersion	35
4	GO	Amine functionalized graphene oxide (AP-GO)^d^	Pd@APGO nanocatalyst	Suzuki and carbonylative Suzuki	Ligand decomposition at acidic pH; moderate toxicity	36
5	GO	—	PdNPs on GO/TiO_2_	Diarylketone synthesis	Difficult Pd dispersion; requires light or high temperature	37
6	Functionalized GO	Sodium ω-carboxyl-*S*-hexanethiosulfate	PdNP/GO or heated PdNP/GO	Suzuki–Miyaura	Possible leaching; sensitivity to conditions	38
7	Graphite oxide	—	Pd^0^ nanoparticles on GO	Suzuki–Miyaura (biaryl)	Relatively low dispersion; requires high temperature	39
**8**	**NA**	**Glucose**	**NA-Fe**_**3**_ **O**_**4**_ **@glucose@Cu–Ni**	**Suzuki and C–S**	**—**	**This work**

a Oxygen reduction reaction.

b 3,6,9-Triaza-1-(2,6)-pyridinacyclodecaphane.

c Graphene nanoplatelets.

d 3-Aminopropyltrimethoxysilane (APTMS).

Therefore, systems based on NA and bio-based ligands like glucose not only reduce environmental concerns, toxicity, and overall cost but, in many cases, demonstrate competitive or even superior performance compared to conventional systems. This approach represents a promising step toward the development of sustainable, low-cost, and environmentally friendly catalysts. The combination of affordable, biodegradable, and accessible raw materials offers an effective strategy for producing green, safe, and economical catalysts that are both simple to prepare and capable of delivering efficient performance in various chemical reactions, serving as a strong alternative to costly and toxic systems.

## 4. Conclusions

In conclusion, we successfully developed a novel magnetic bimetallic Cu–Ni catalyst supported on NA using glucose as a biocompatible and non-toxic ligand. This green catalytic system demonstrated outstanding activity and chemoselectivity in the synthesis of asymmetric biaryls and sulfides under mild conditions, achieving high yields (up to 99%) and remarkable recyclability in 6 consecutive runs with negligible metal leaching. The use of NA as a low-cost, abundant, and chemically rich support, combined with glucose as a stabilizing ligand, effectively prevented nanoparticle agglomeration and ensured structural integrity. This work highlights the advantages of employing natural and eco-friendly materials over conventional, expensive, and toxic carbon-based supports and ligands. The developed catalytic system not only aligns with green chemistry principles but also offers a cost-effective and environmentally benign alternative with competitive catalytic performance. Therefore, the integration of NA and glucose-based ligands represents a promising strategy for designing sustainable, reusable, and efficient catalysts for diverse chemical transformations, underscoring its potential for scalable industrial applications.

## Author contributions

Sahar Abdolahi: writing – review & editing, writing – original draft, methodology, validation, resources, conceptualization, investigation, formal analysis, data curation. Mohammad Soleiman-Beigi: supervision, writing – review & editing, conceptualization, resources, project administration.

## Conflicts of interest

The authors declare no competing financial interest.

## Supplementary Material

NA-OLF-D5NA00899A-s001

## Data Availability

All data generated or analyzed during this study are included in this published article [and its supplementary information (SI)]. Supplementary information (SI) is available. See DOI: https://doi.org/10.1039/d5na00899a.
