# Supplementary material for: Sustainable bimetallic Cu/Ni catalysts: leveraging glucose for enhanced immobilization on magnetic Fe3O4/amino natural asphalt composites in coupling reactions
Source: Nanoscale Adv. 2025 Dec 2;8(1):271–85. doi: 10.1039/d5na00899a (PMC12679291; doi:10.1039/d5na00899a)
Supplement: NA-008-D5NA00899A-s001 [file NA-008-D5NA00899A-s001.pdf]

Supplementary Information (SI) for Nanoscale Advances.
This journal is © The Royal Society of Chemistry 2025

Supporting Information

Sustainable Bimetallic Cu/Ni Catalysts: Leveraging Glucose for Enhanced Immobilization on Magnetic Fe_3O_4/Amino Natural Asphalt Composites in Coupling Reactions

Sahar Abdolahi [a], Mohammad Soleiman-Beigi [a,*]

[*] [a] Department of Chemistry, Faculty of Basic Sciences, Ilam University, P.O. Box 69315516, Ilam, Iran.
E-mail: *SoleimanBeigi@yahoo.com*; *m.soleimanbeigi@ilam.ac.ir*

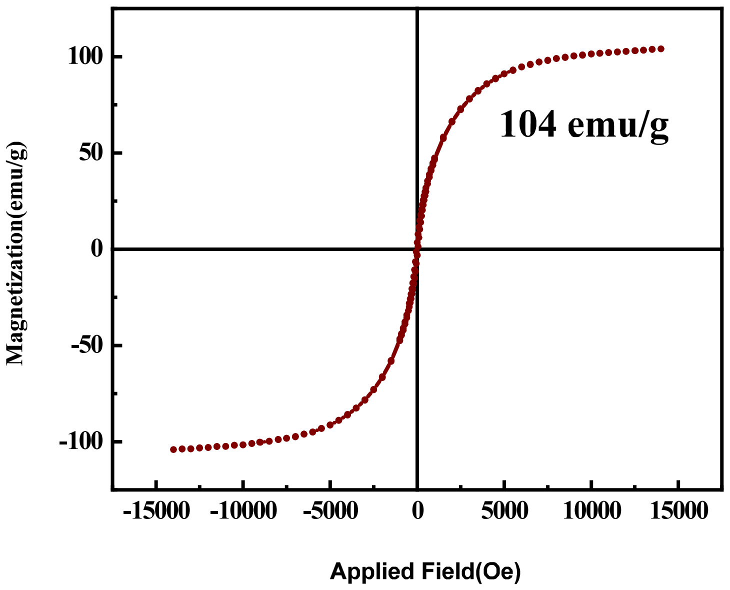

Fig. S1 VSM diagram of Fe_3O_4@NA-NH_2

In the interpretation of the infrared spectrum of D-glucose, the most prominent absorption bands appear in the wavenumber range from approximately 2500 to 3700 cm^{-1}, consisting of two broad overlapping bands due to O-H and C-H stretching vibrations. These bands are complicated by hydrogen bonding, which affects the O-H vibration frequencies, and the O-H vibrations can originate from both primary and secondary alcohol groups. Absorptions in the region of 3200 to 3500 cm^{-1} are mainly attributed to O-H stretching vibrations, with twin peaks possibly arising from both primary and secondary alcohol groups, while absorptions between 2800 and 3000 cm^{-1} are primarily due to C-H stretching vibrations. The C-O stretching vibrations of the ether C-O-C group occur around 1070 to 1140 cm^{-1}, whereas C-O vibrations from a primary alcohol group typically appear between 1000 and 1075 cm^{-1}, and those from a secondary alcohol group occur around 1075 to 1150 cm^{-1}.

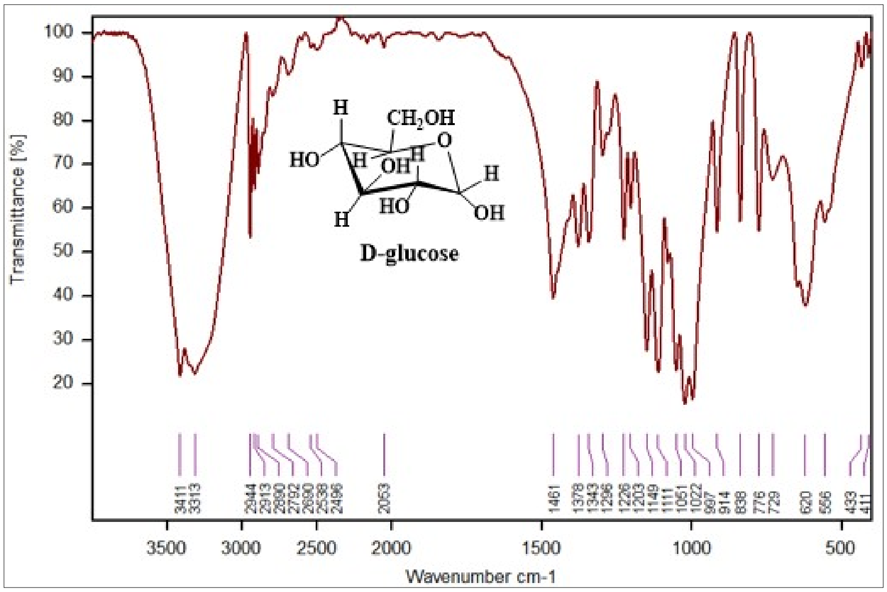

Fig. S2 FT-IR of D-glucose

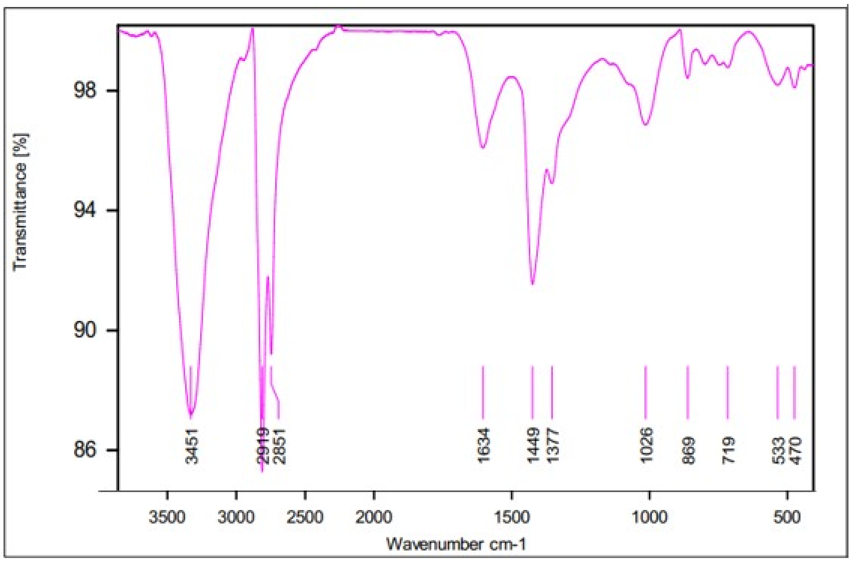

Fig. 3 FT-IR spectrum of NA

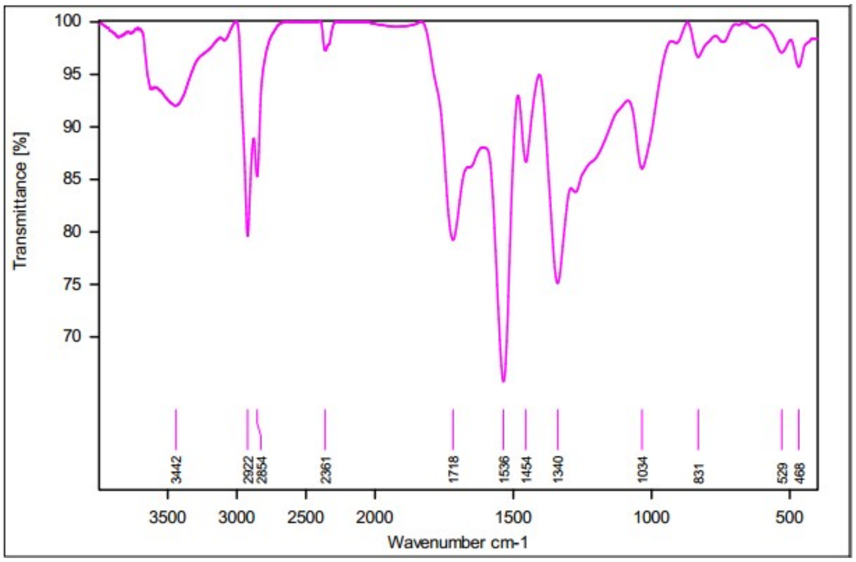

Fig. 4 FT-IR spectrum of NA-NO_2

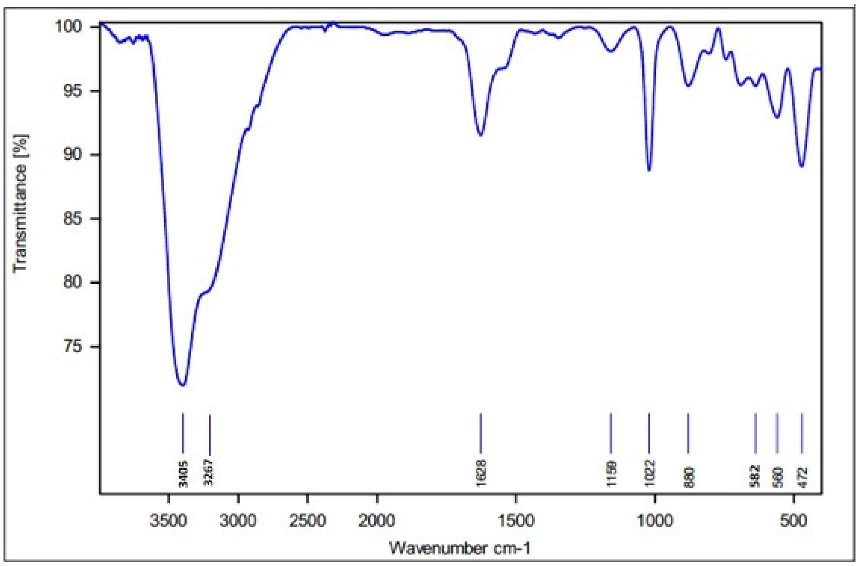

Fig. 5 FT-IR spectrum of Fe_3O_4@NA-NH_2

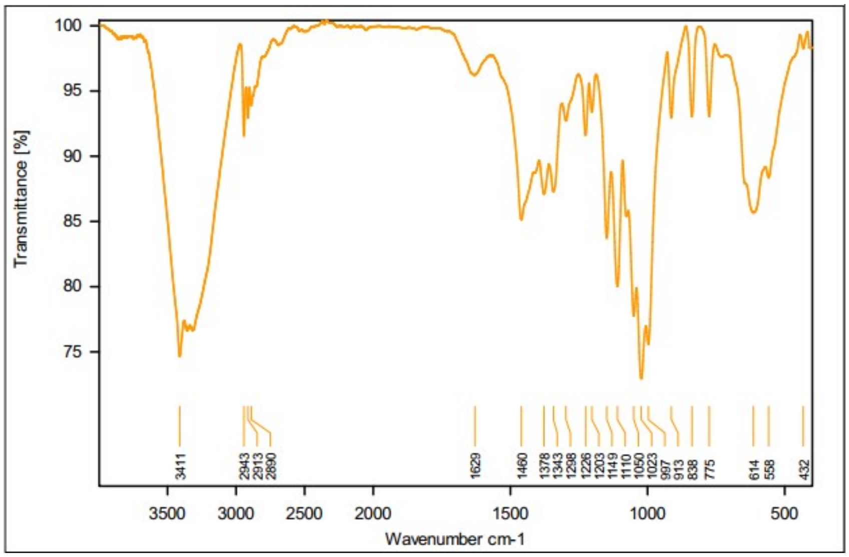

Fig. 6 FT-IR spectrum of NA-Fe_3O_4@glucose

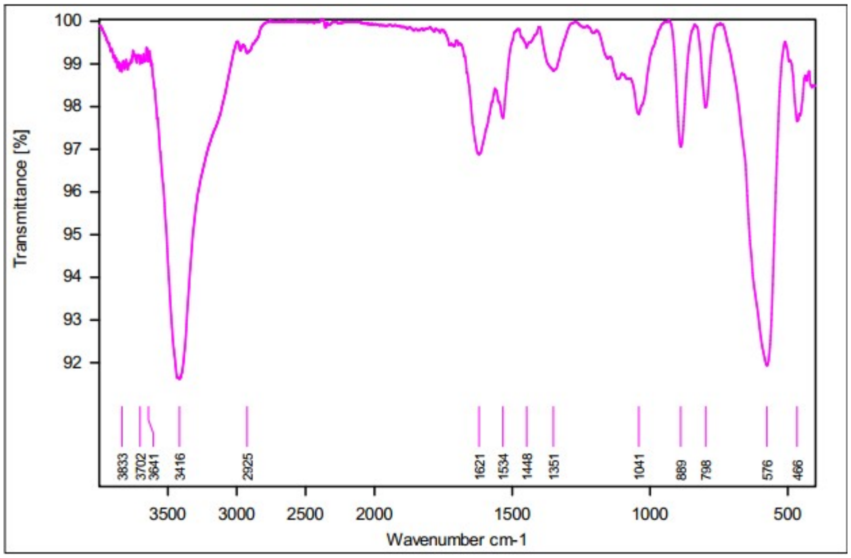

Fig. 7 FT-IR spectrum of NA-Fe_3O_4@glucose@Cu-Ni

Table S1 indicates the standard reference files for the XRD peaks of the ferrite phases Fe_3O_4, $NiFe_2O_4$, and $CuFe_2O_4$. These reference files provide the characteristic peak positions and crystal structures that are used for phase identification in XRD analysis. Fe_3O_4, and $NiFe_2O_4$ are well-studied and widely referenced, with their peaks typically observed around specific 2θ angles. $CuFe_2O_4$ is less commonly documented but exhibits similar peak patterns due to its similar cubic spinel structure.

Table S1 Standard reference files for XRD peaks.

**Phase**	**Standard reference number (JCPDS/ICDD)**	**Crystal structure**	**Details**
Fe_3O_4 **(Magnetite)**	19-0629	Cubic (Fd-3m)	Main peaks around 30°, 35°, 43°, 53°, 57°, 62°
$NiFe_2O_4$ **(Nickel Ferrite)**	10-0325	Cubic (Fd-3m)	Similar peak positions to Fe_3O_4, with slight differences
$CuFe_2O_4$ **(Copper Ferrite)**	11-0835	Cubic (Fd-3m)	Less common in references, but has similar peaks to other ferrites

Table S2 Optimization of different base amounts for the synthesis of 1,1′-biphenyl using NA-Fe_3O_4@glucose@Cu-Ni**.**

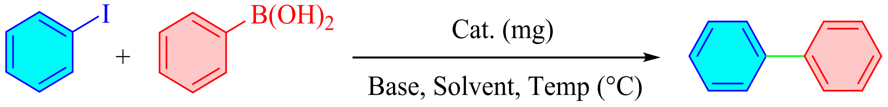

3a

Entry	Base	Amount of base (mmol)	Temperature (°C)	Solvent	Amount of catalyst (mg)	Yield (%)[b]
**1**	**K_2CO_3**	**1**	**80**	**PEG**	**10**	**99**
2	K_2CO_3	2	80	PEG	10	99
3	K_2CO_3	3	80	PEG	10	98
4	Ca_2CO_3	1	80	PEG	10	93
5	Ca_2CO_3	2	50	PEG	10	94
6	Ca_2CO_3	3	80	PEG	10	93
7	Na_2CO_3	1	80	PEG	10	94
8	Na_2CO_3	2	80	PEG	10	95
9	Na_2CO_3	3	80	PEG	10	94
10	Cs_2CO_3	1	80	PEG	10	90
11	Cs_2CO_3	2	80	PEG	10	91
12	Cs_2CO_3	3	80	PEG	10	91
13	NaOH	1	80	PEG	10	91
14	NaOH	2	80	PEG	10	93
15	NaOH	3	80	PEG	10	92
16	KOH	1	80	PEG	10	92
17	KOH	2	80	PEG	10	94
18	KOH	3	80	PEG	10	93

Table S3 Optimization of different base amounts for the synthesis of sulfides using NA-Fe_3O_4@glucose@Cu-Ni.

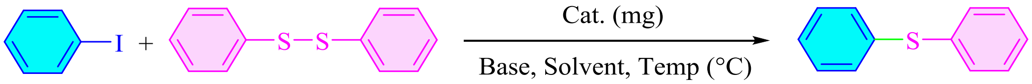

5a

Entry	Base	Amount of base (mmol)	Temperature (°C)	Solvent	Amount of catalyst (mg)	Yield (%)[b]
**1**	KOH	**1**	**80**	**PEG**	**20**	**98**
2	KOH	2	80	PEG	20	98
3	KOH	3	80	PEG	20	97
4	Cs_2CO_3	1	80	PEG	20	90
5	Cs_2CO_3	2	50	PEG	20	91
6	Cs_2CO_3	3	80	PEG	20	91
7	NaOH	1	80	PEG	20	97
8	NaOH	2	80	PEG	20	97
9	NaOH	3	80	PEG	20	96
10	Na_2CO_3	1	80	PEG	20	91
11	Na_2CO_3	2	80	PEG	20	92
12	Na_2CO_3	3	80	PEG	20	91
13	K_2CO_3	1	80	PEG	10	92
14	K_2CO_3	2	80	PEG	10	92
15	K_2CO_3	3	80	PEG	10	91

Spectral data of some of the products:

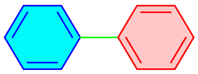

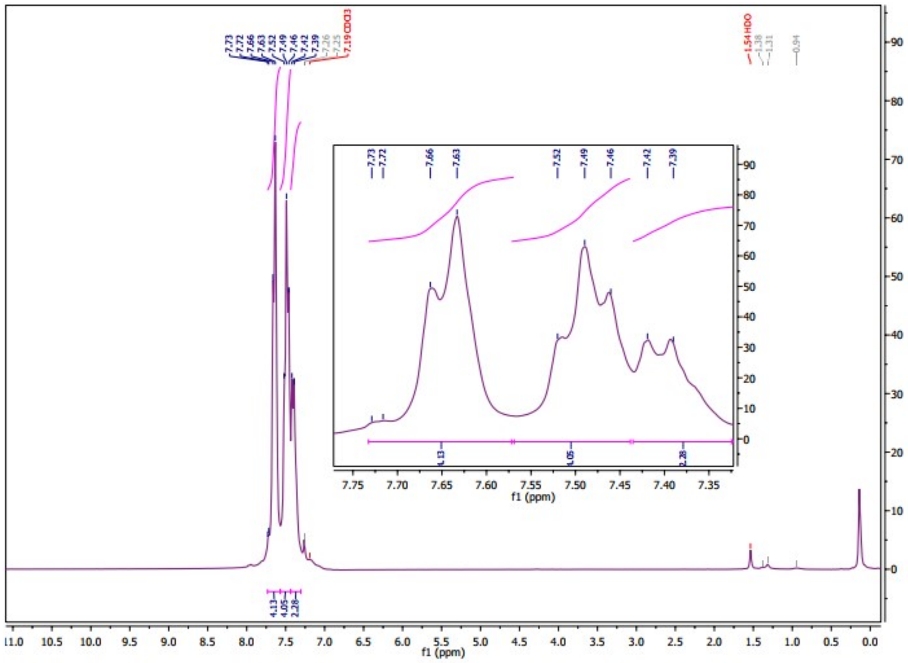

Fig. S8 ^{1}H NMR of 1,1'-biphenyl

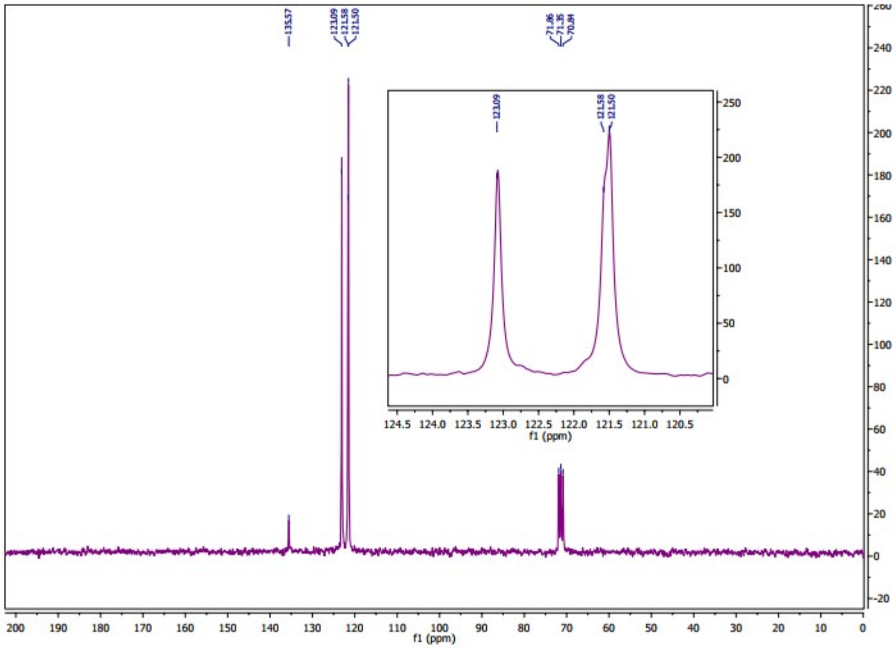

Fig. S9 ^{13}C NMR of 1,1'-biphenyl

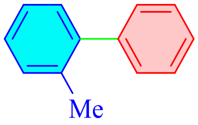

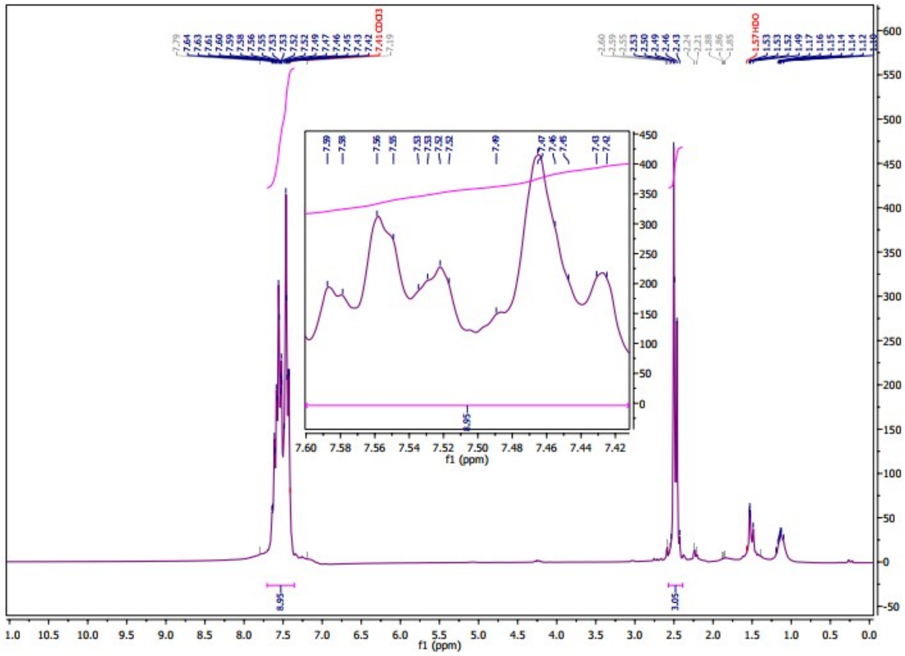

Fig. S10 ^{1}H NMR of 2-methyl-1,1'-biphenyl

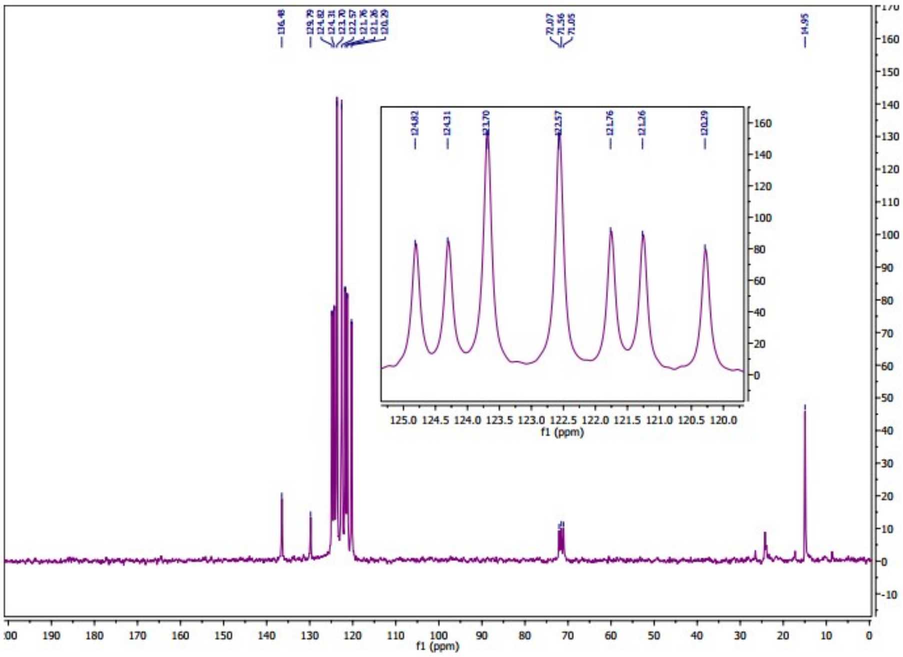

Fig. S11 ^{13}C NMR of 2-methyl-1,1'-biphenyl

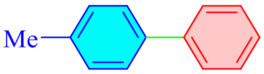

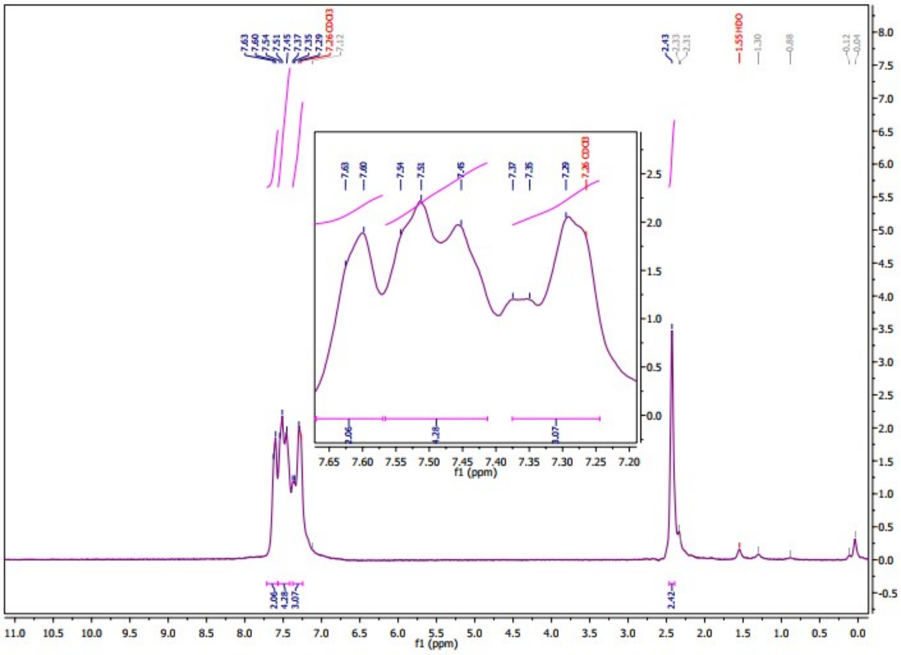

Fig. S12 ^{1}H NMR of 4-methyl-1,1'-biphenyl

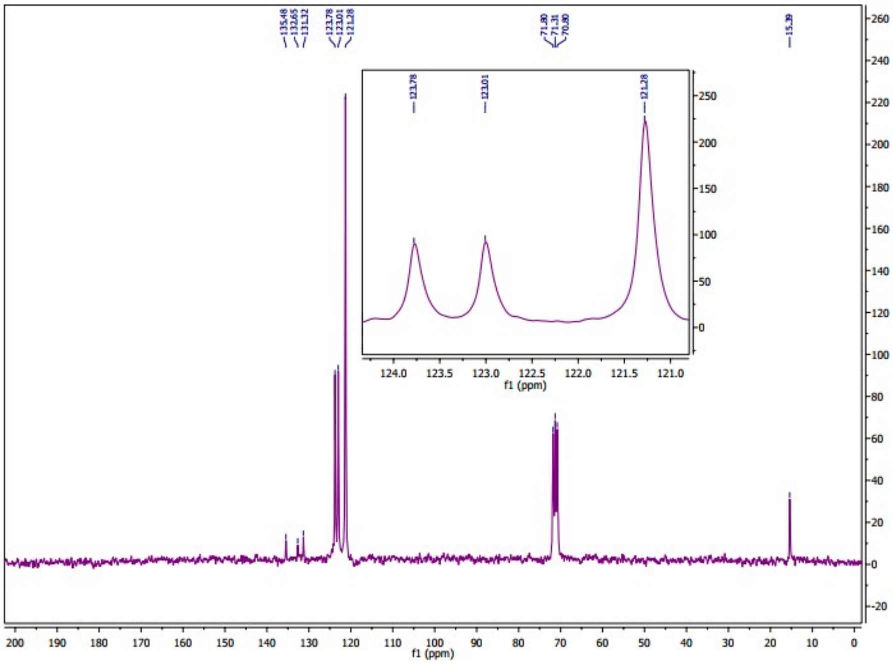

Fig. S13 ^{13}C NMR of 4-methyl-1,1'-biphenyl

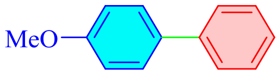

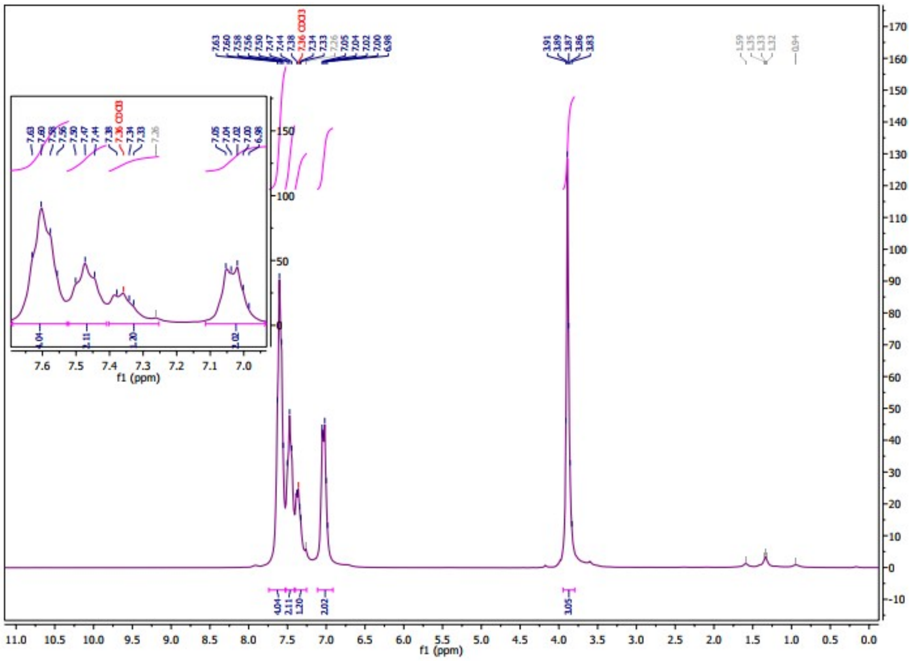

Fig. S14 ^{1}H NMR of 4-methoxy-1,1'-biphenyl

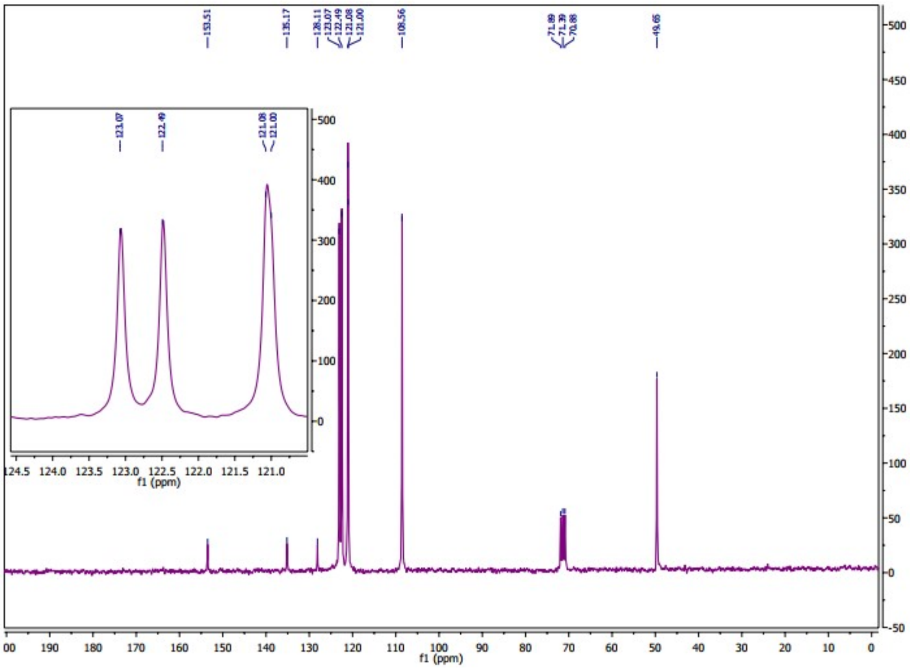

Fig. S15 ^{13}C NMR of 4-methoxy-1,1'-biphenyl

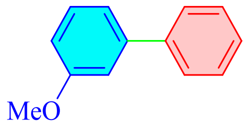

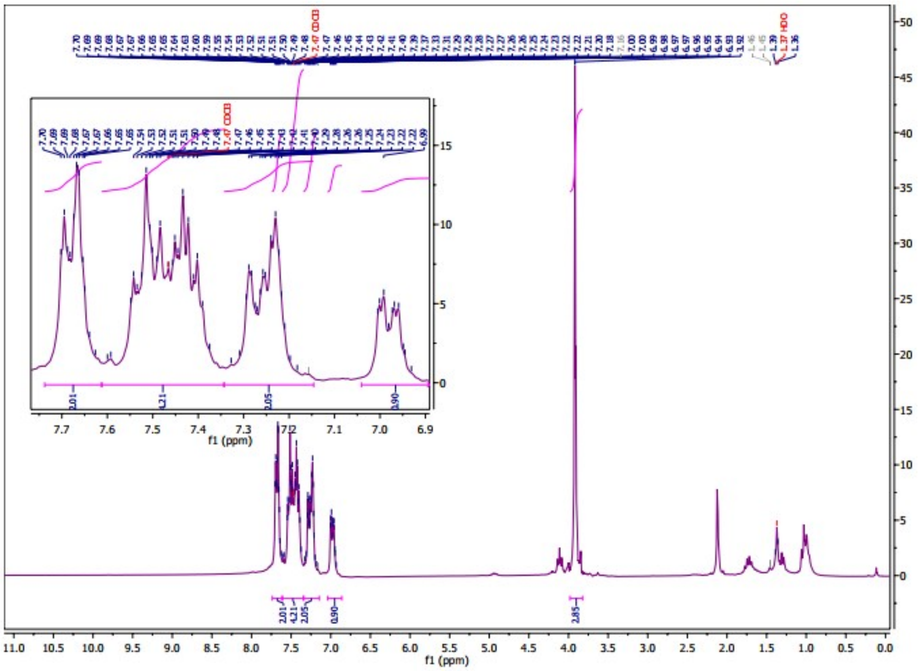

Fig. S16 ^{1}H NMR of 3-methoxy-1,1'-biphenyl

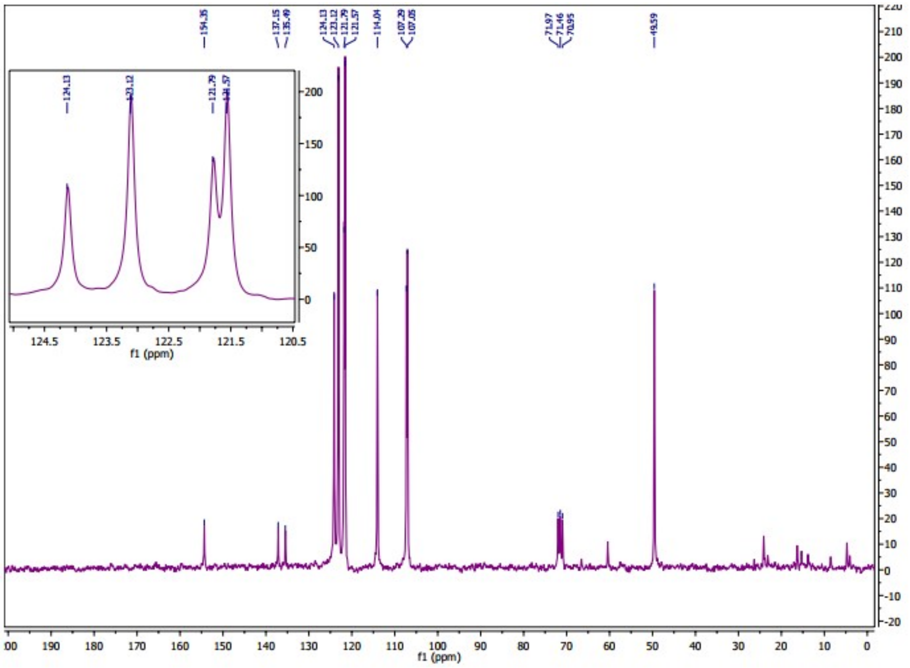

Fig. S17 ^{13}C NMR of 3-methoxy-1,1'-biphenyl

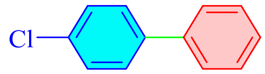

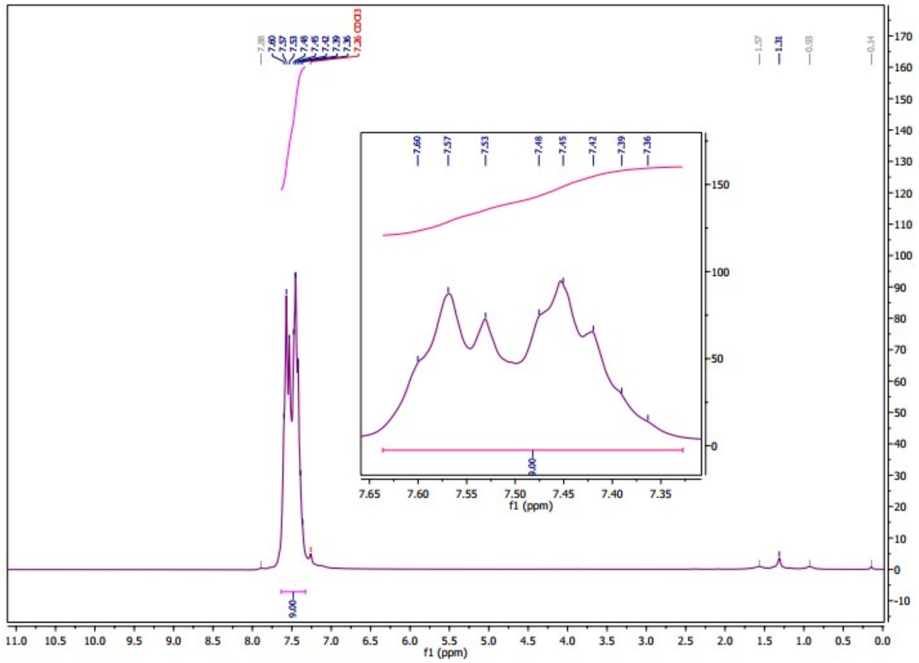

Fig. S18 ^{1}H NMR of 4-chloro-1,1'-biphenyl

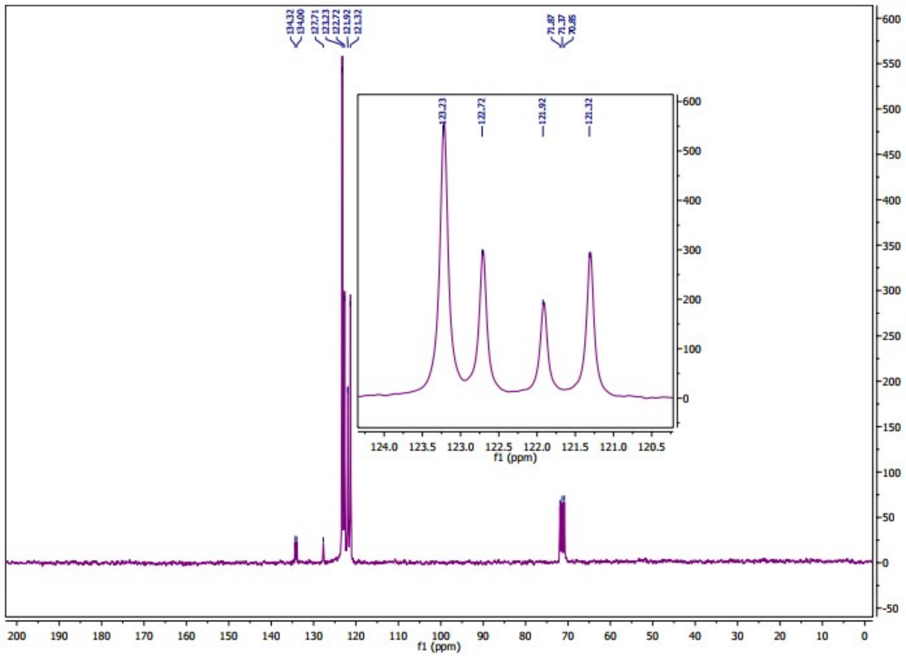

Fig. S19 ^{13}C NMR of 4-chloro-1,1'-biphenyl

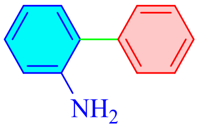

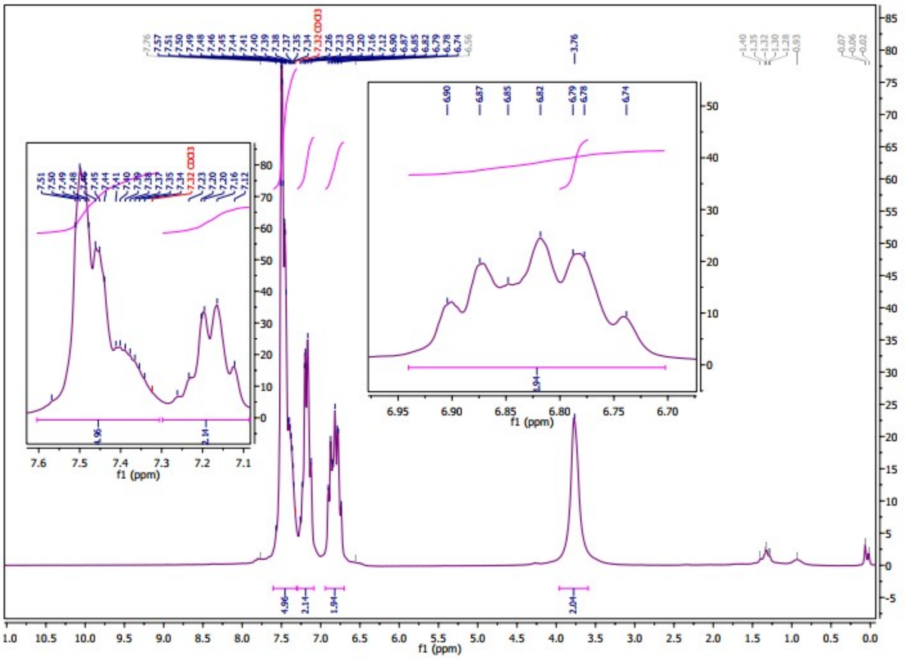

Fig. S20 ^{1}H NMR of 2-Aminobiphenyl

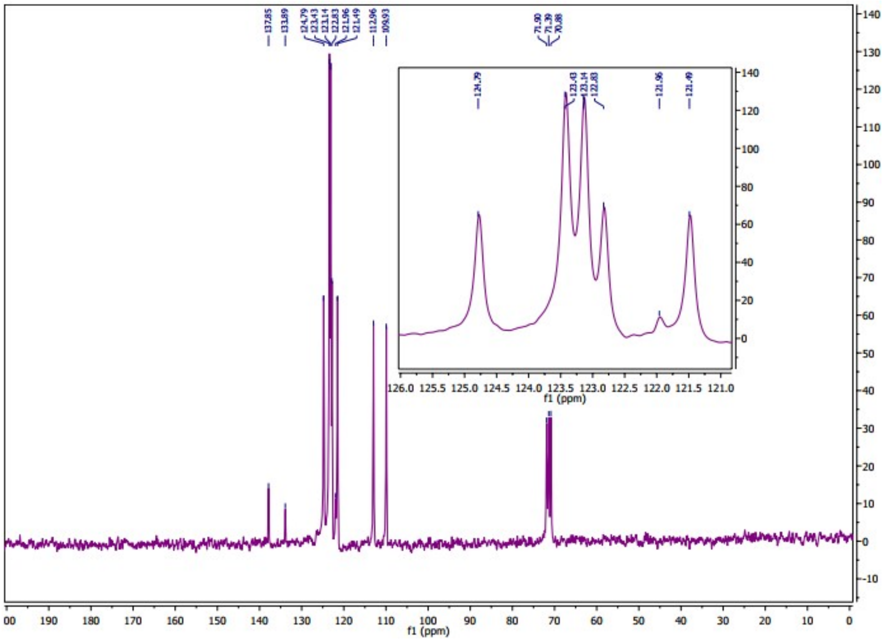

Fig. S21 ^{13}C NMR of 2-Aminobiphenyl

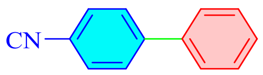

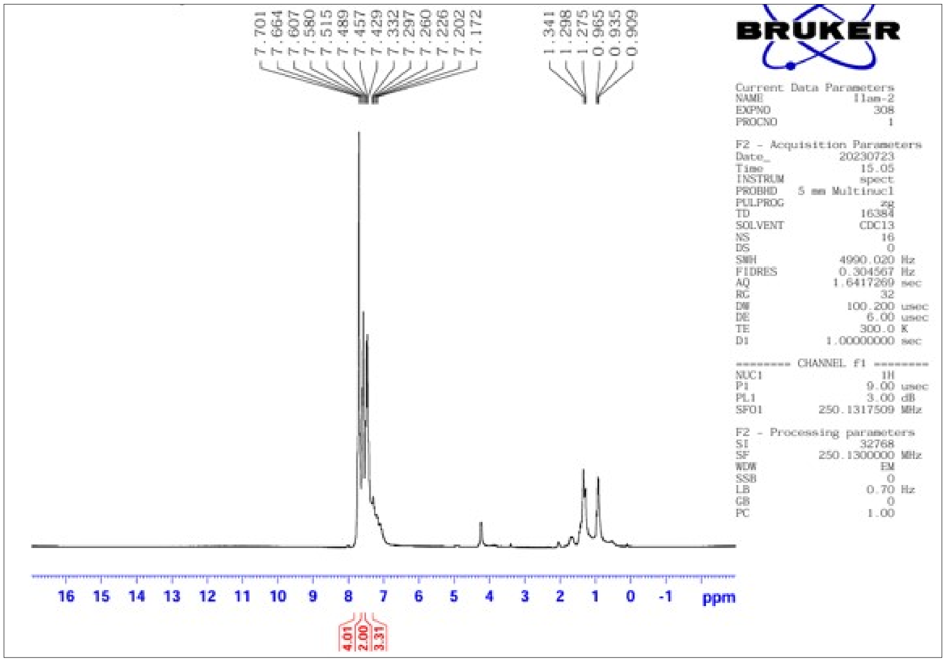

Fig. S22 ^{1}H NMR of 4-cyano-1,1'-biphenyl

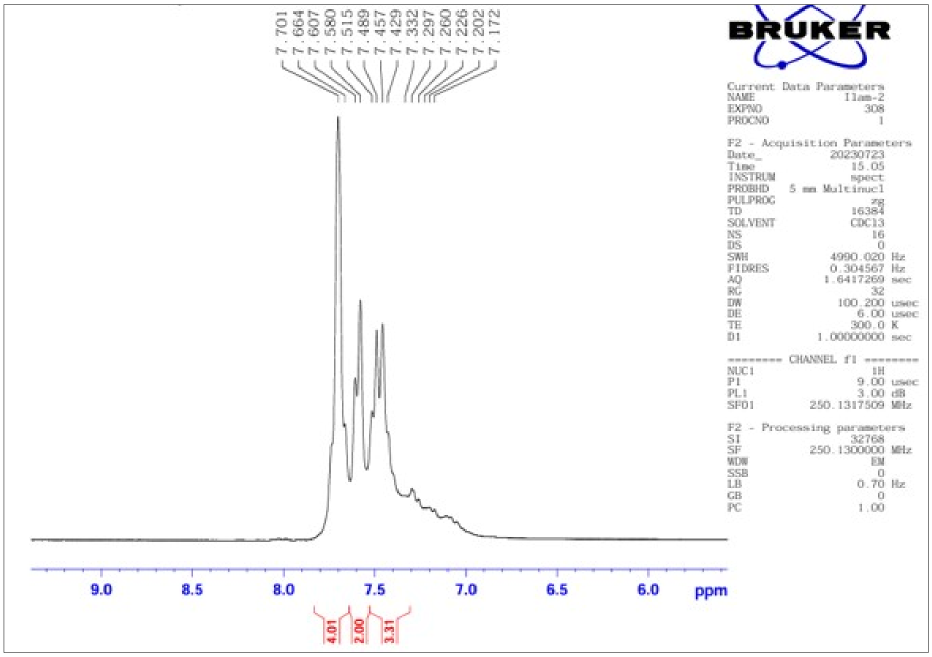

Fig. S23 ^{1}H NMR of 4-cyano-1,1'-biphenyl (Expand)

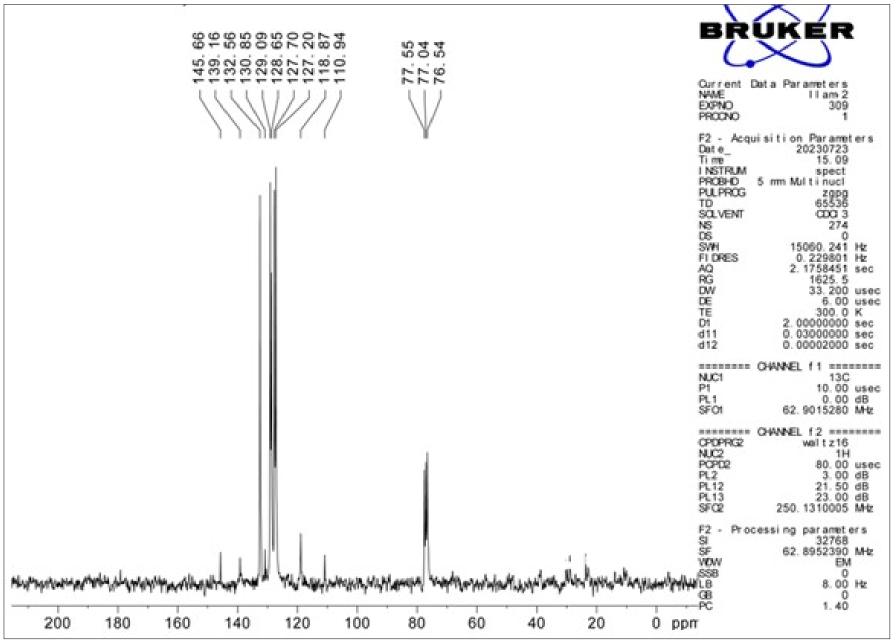

Fig. S24 ^{13}C NMR of 4-cyano-1,1'-biphenyl

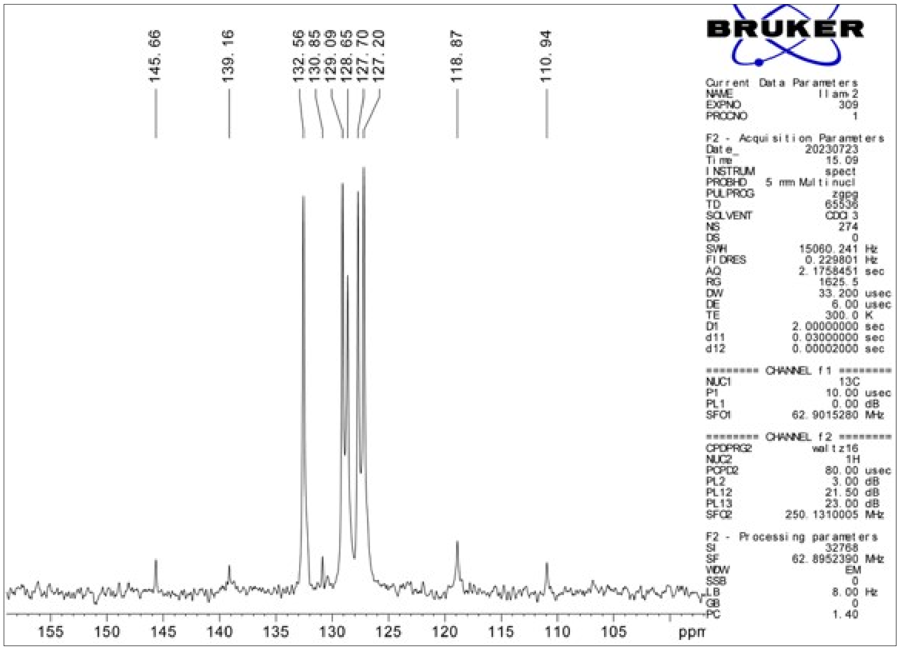

Fig. S25 ^{13}C NMR of 4-cyano-1,1'-biphenyl (Expand)

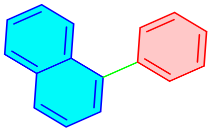

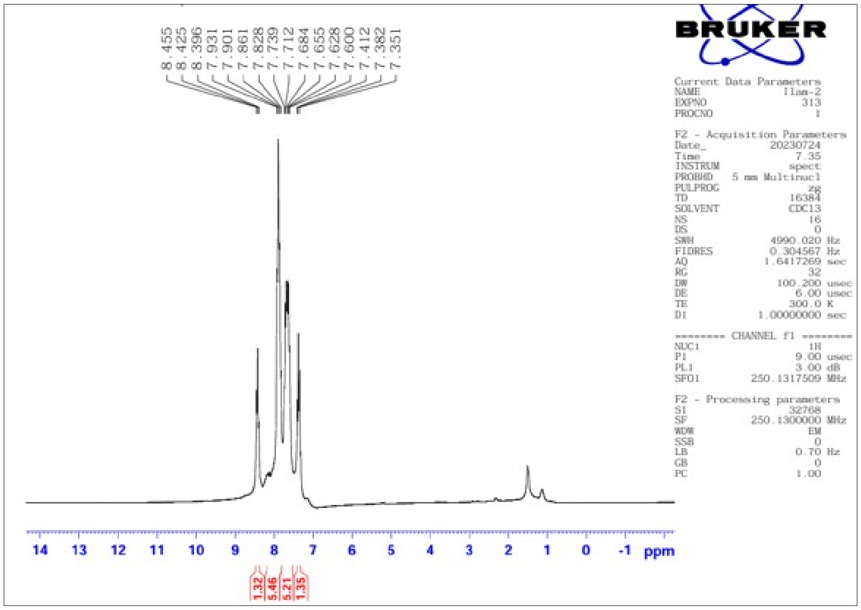

Fig. S26 ^{1}H NMR of 1-phenylnaphthalene

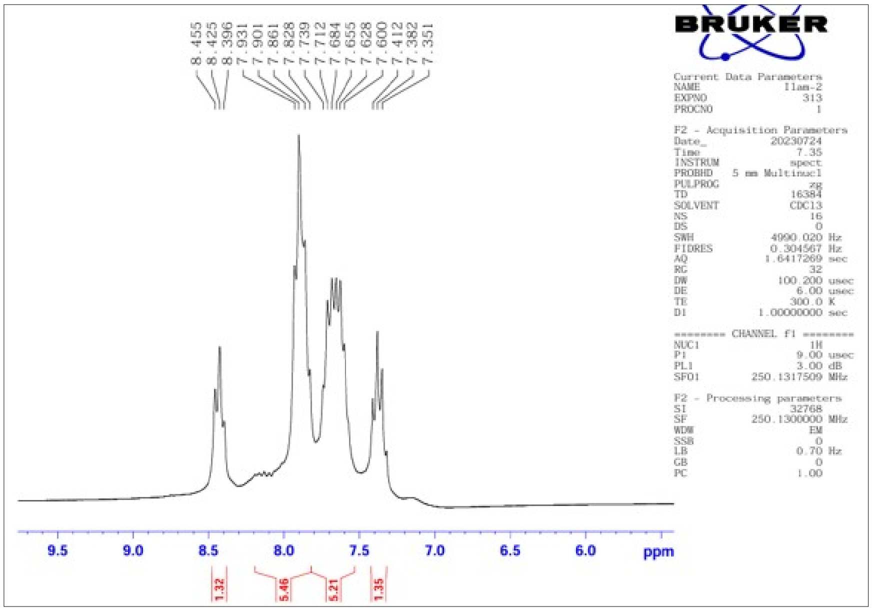

Fig. S27 ^{1}H NMR of 1-phenylnaphthalene (Expand)

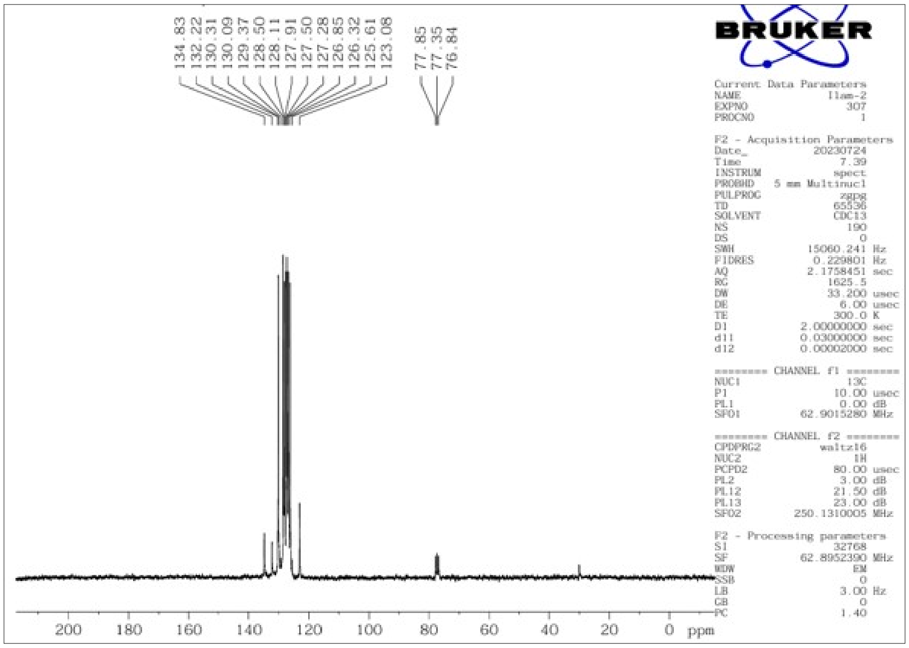

Fig. S28 ^{13}C NMR of 1-phenylnaphthalene

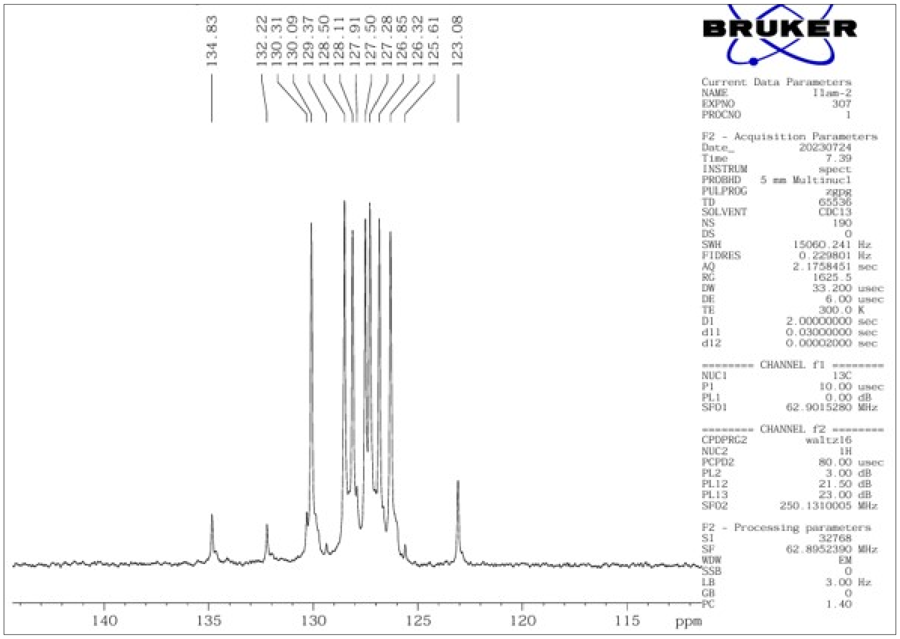

Fig. S29 ^{13}C NMR of 1-phenylnaphthalene (Expand)

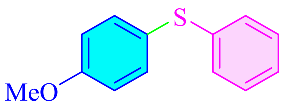

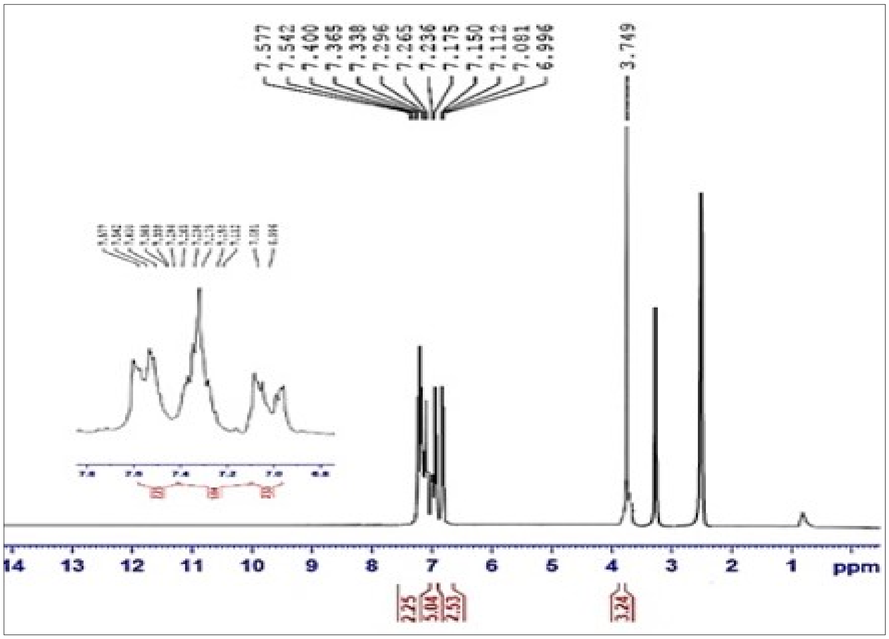

Fig. S30 ^{1}H NMR of (4-methoxyphenyl)(phenyl)sulfide

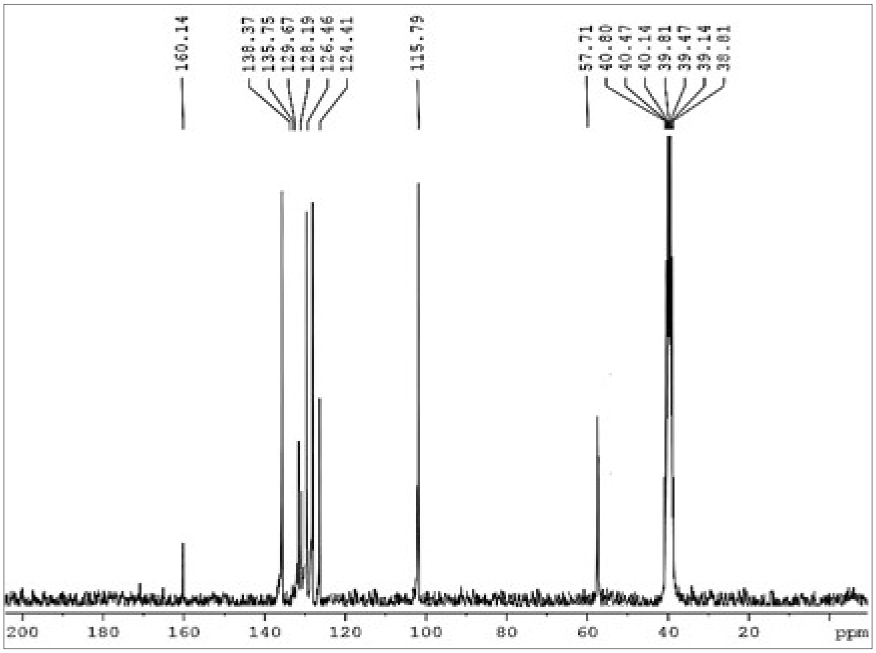

Fig. S31 ^{13}C NMR of (4-methoxyphenyl)(phenyl)sulfide

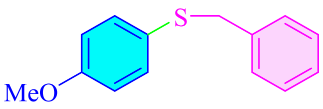

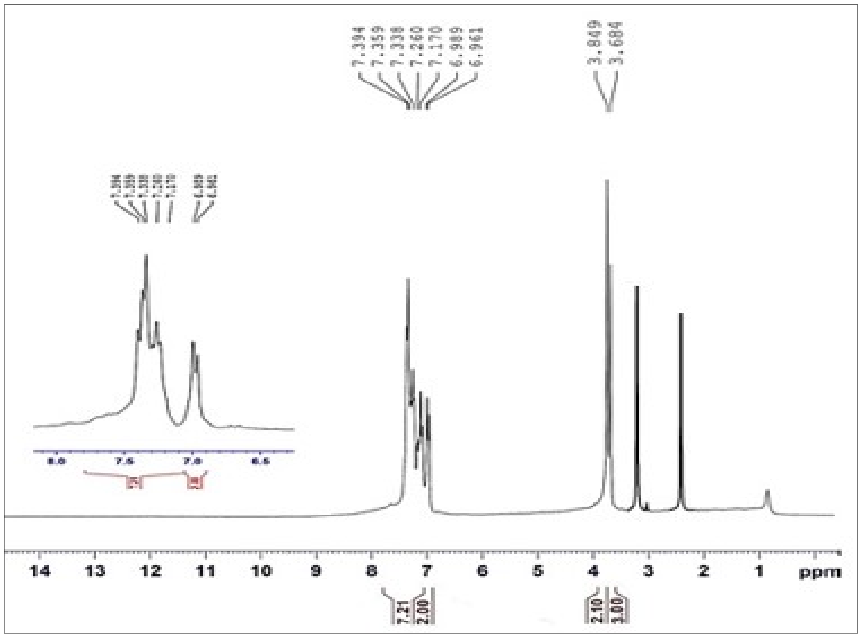

Fig. S32 ^{1}H NMR of benzyl(4-methoxyphenyl)sulfane

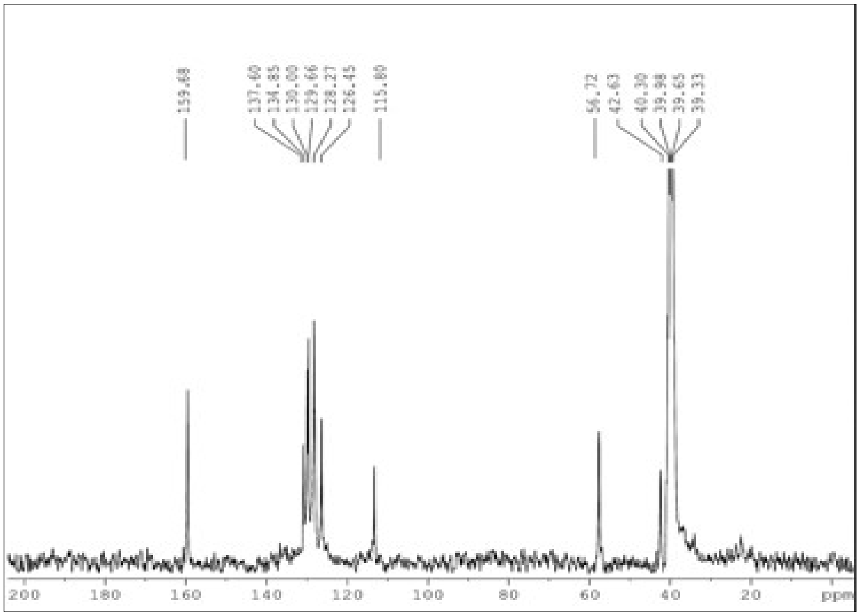

Fig. S33 ^{13}C NMR of benzyl(4-methoxyphenyl)sulfane
